# Therapeutic effects of curcumin on upper gastrointestinal diseases: a systematic review and meta-analysis of animal studies

**DOI:** 10.1186/s12906-025-05175-4

**Published:** 2025-12-05

**Authors:** Kankanit Yeerong, Ratchanon Inpan, Jakkrit Aisara, Kornvipa Settakorn, Mingkwan Na Takuathung, Nithi Thinrungroj, Nut Koonrungsesomboon

**Affiliations:** 1 Clinical Research Center for Food and Herbal Product Trials and Development (CR-FAH), Faculty of Medicine, Chiang Mai University, Chiang Mai, 50200 Thailand; 2 Department of Pharmacology, Faculty of Medicine, Chiang Mai University, Chiang Mai, 50200 Thailand; 3 Office of Research Administration, Chiang Mai University, Chiang Mai, 50200 Thailand; 4 Division of Gastroenterology, Department of Internal Medicine, Faculty of Medicine, Chiang Mai University, Chiang Mai, 50200 Thailand

**Keywords:** Curcumin, Diabetic gastroparesis, Gastric ulcer, Gastritis, Helicobacter pylori, Peptic ulcer, Upper gastrointestinal tract

## Abstract

**Background:**

Curcumin, the main bioactive compound in *Curcuma longa* L., has long been traditionally used for the treatment of upper gastrointestinal diseases. Preclinical studies have provided substantial evidence supporting its potential therapeutic effects. However, a systematic synthesis of these findings is lacking. This study aimed to evaluate the effects of curcumin on upper gastrointestinal outcomes in animals through systematic review and meta-analysis.

**Methods:**

Studies were retrieved from three databases (PubMed, Scopus, and Embase) and through a hand search in Google Scholar, covering the period from inception to 2024. Animal studies comparing curcumin with control were selected and analyzed using RStudio version 2024.12.0. A random-effects model was applied to estimate the main effect sizes. The risk of bias was assessed using the SYRCLE’s tool. Subgroup analysis, meta-regression, and qualitative synthesis were also performed.

**Results:**

A total of 49 studies were included in the analysis. The results indicated that curcumin significantly improved various outcomes, such as acid output (SMD = -1.47; 95% CI: -2.03–-0.92; *p* < 0.0001) and ulcer index (SMD = -2.19; 95% CI: -2.64–-1.73; *p* < 0.0001) in animals. It also improved oxidative stress, inflammatory markers, blood glucose level, and gastric emptying rate. However, the analyses revealed substantial heterogeneity, and the risk of bias was unclear in most studies, with five studies assessed as having a high risk of bias.

**Conclusions:**

Curcumin exhibits beneficial effects on various upper gastrointestinal disorders in animal models, including gastric ulcers, diabetic gastroparesis, esophageal diseases, *Helicobacter pylori* infection, and gastritis. These findings underscore curcumin’s therapeutic potential and highlight the need for clinical investigations to validate its translational applicability in humans.

**Systematic review registration:**

Registration with the International Prospective Register of Systematic Reviews (PROSPERO) (registration number: CRD42024603162).

**Supplementary Information:**

The online version contains supplementary material available at 10.1186/s12906-025-05175-4.

## Background

Upper gastrointestinal (GI) diseases, such as peptic ulcer disease, gastritis, esophagitis, and gastroesophageal reflux disease (GERD), are highly prevalent and significantly impact both patient health and healthcare systems globally [1, 2]. These diseases may result in serious complications, such as gastrointestinal bleeding and perforation, whose incidence increases with age and is nearly twice as high in men as in women [3]. Despite the widespread use of standard pharmacological therapies such as proton pump inhibitors (PPIs), histamine (H_2_)-receptor antagonists, and antacids, many patients still experience incomplete symptom resolution [4]. This imperfect response can be attributed to the complex interplay of various pathogenic factors, ranging from *Helicobacter pylori *infection, low-grade inflammation, visceral hypersensitivity, and delayed gastric emptying [5, 6]. Consequently, it often necessitates prolonged pharmacological therapy and raises important safety concerns; for instance, long-term PPI use has been linked to acute interstitial nephritis, bone fractures, poor COVID-19 infection outcomes, and pneumonia [7]. As a result, there is a growing interest in exploring alternative or adjunctive treatments, including herbal products and dietary supplements, which provide additional therapeutic benefits by targeting diverse mechanisms underlying upper GI pathology and offering a favorable safety profile.

Curcumin (diferuloylmethane), the primary bioactive compound derived from *Curcuma longa *L. (turmeric), has a long history of use in traditional and Ayurvedic medicine, particularly in India and China [8]. It is the most abundant substance of the curcuminoid family, constituting approximately 60–70% of the total curcuminoid content [9]. Due to its potent gastroprotective, anti-inflammatory, antioxidant, and antimicrobial properties, curcumin has been widely employed to treat a variety of GI diseases, including indigestion, flatulence, diarrhea, and peptic ulcers [10]. However, the lack of clearly defined therapeutic dosage limits its broader clinical applicability. According to the Joint FAO/WHO Expert Committee on Food Additives (JECFA), the acceptable daily intake (ADI) of curcumin is 0–3 mg per kilogram of body weight per day, based on a no-observed-effect level (NOEL) derived from animal studies [11]. The European Food Safety Authority (EFSA) has similarly emphasized adherence to this ADI when curcumin is used as a food additive [12]. In Thailand, turmeric rhizome powder used in solid dosage formulations is listed in the national list of essential herbal medicines and is indicated for the relief of symptoms such as bloating, indigestion, and flatulence; however, the curcumin content of these preparations is not standardized [13]. Hence, concerns regarding the safety of curcumin have been raised. The Italian Ministry of Health has prohibited all health claims for turmeric supplements and mandated cautionary labeling in response to reports of liver injury associated with high-bioavailability curcumin formulations, particularly those combined with piperine [14]. Consequently, establishing robust preclinical evidence is essential for providing foundational insights into its therapeutic potential, as such investigations allow controlled conditions, standardized dosing, evaluation of mechanistic pathways, and clarification of dose–response relationships. These insights are critical for guiding the design and interpretation of rigorous clinical studies. However, differences between species, such as metabolic rates, immune responses, and receptor expression, remain an important concern. Therefore, this study aimed to synthesize available evidence on the effects of curcumin in animal models of upper GI disorders through a systematic review and meta-analysis, thereby contributing to a more comprehensive understanding of its therapeutic potential.

## Methods

### Literature search

This study protocol adhered to the Preferred Reporting Items for Systematic Reviews and Meta-Analysis Protocol (PRISMA-P) 2015 statement and was registered with the International prospective register of systematic reviews (PROSPERO) (registration number: CRD42024603162). This study has been granted an exemption certificate by the Research Ethics Committee of the Faculty of Medicine, Chiang Mai University (EXEMPTION 0629/2024). Additionally, this study is reported in compliance with the PRISMA 2020 checklist.

### Search strategies and literature search

A comprehensive search was conducted in PubMed, Embase, and Scopus, in November 2024. The search strategy was based on the approach used in previous articles to ensure comprehensive search [15, 16]. The complete search strings for each database are provided in Supplementary Table 1. The population included terms related to upper GI diseases and conditions (e.g., gastric ulcer, gastritis, esophagitis, dyspepsia, GERD, *H. pylori* infection). For the intervention search, terms including curcumin, curcuma, curcuminoids, turmeric, and diferuloylmethane were used. The ‘OR’ operator was used to combine terms within the same domain, while the two domains were combined using the ‘AND’ operator. In addition to the database search, Google Scholar was searched as part of a hand search to identify additional studies and grey literature. Reference lists of full-text articles were also screened to identify further relevant publications. The search was limited to articles published up to 2024, and no language restrictions were applied.

### Study selection

All records retrieved through the search strategy were screened, and any duplicates were removed prior to title and abstract screening. Two review authors (K.Y. and R.I.) independently screened titles and abstracts of the remaining articles and assessed the eligibility of full-text articles. The eligibility criteria were defined according to the PICOS (Population, Intervention, Comparison, Outcomes, and Study design) framework, as summarized in Table 1. In vivo studies comparing curcumin with control, including a placebo or vehicle control, on pharmacodynamic outcomes related to upper GI diseases and conditions were selected. Only studies that specified the curcumin content, including those using standardized formulations or extracts, were included.

**Table 1:** PICOS framework for eligibility criteria

PICOS	Description
P	Animals with experimentally induced upper gastrointestinal diseases
I	Turmeric or curcumin with clearly defined curcumin content, including standardized extracts or formulations
C	Vehicle or normal saline solution.
O	Outcomes related to upper gastrointestinal diseases, such as biochemical markers, gastric ulcer parameters, gastric acid parameters, diabetic gastroparesis outcomes
S	In vivo animal studies with controlled experimental design

*Abbreviation*: *C *comparison, *I *intervention, *O* outcomes, *P* population, *S* study design

Studies were excluded if they focused only on pharmacokinetics, were in vitro experiments, used curcumin combined with other active ingredients, reported only qualitative pathological findings, did not report the number of animals, did not specify the curcumin content, or involved curcumin analogs or derivatives. Review articles, case reports, meta-analyses, conference proceedings and abstracts, book chapters, short communications, and expert opinions were also excluded from this study. Any disagreements between the two review authors were resolved through discussion and consensus, and, if necessary, by consulting with a third reviewer (N.K.). 

### Data extraction

Two review authors (K.Y. and R.I.) independently extracted the following data using a data extraction form: first author, year of publication, population, number of animals, induction method, characteristics of the intervention and control, treatment duration, and pharmacodynamic GI-related outcomes. Outcomes for gastric ulcer studies were categorized into three domains: (1) biochemical markers (e.g. catalase (CAT), superoxide dismutase (SOD), glutathione (GSH), malondialdehyde (MDA), inducible nitric oxide synthase (iNOS), and tumor necrosis factor-alpha (TNF-α) levels); (2) gastric acid parameters (e.g. acid output, gastric acid volume, gastrin level, hydrogen potassium adenosine triphosphatase (H⁺/K⁺ ATPase) level, potential of hydrogen (pH) of gastric acid, and total acidity); (3) gastric ulcer parameters (e.g. ulcer area, ulcer index, mucosal healing index, and ulcer healing index). Diabetic gastroparesis outcomes included blood glucose level and gastric emptying rate. Studies with multiple induction methods, administration routes, or animal species were considered separately. When articles reported ranges of animal numbers, selected values were used for sensitivity analysis. If curcumin was incorporated into a delivery system, the equivalent curcumin dose was used. However, if the equivalent dose was not provided, it was calculated based on the encapsulation efficiency. Data from studies with multiple curcumin dosages were combined for comparison with controls using the StatsToDo website. When articles presented data only in graphical form, WebPlotDigitizer was used to extract numerical values for analysis. If data in the original publication were missing or unclear, the corresponding authors were contacted via email for clarification or additional details. Studies for which the necessary information could not be obtained were subsequently excluded from the analysis. Any discrepancies or inconsistencies were resolved through discussion and consensus, and, if necessary, by consulting with a third reviewer (N.K.).

### Risk of bias assessment

The risk of bias (RoB) in animal experiments was independently assessed by two reviewers (K.Y. and R.I.) using the SYRCLE’s RoB tool [17], with discrepancies resolved through discussion, and, if necessary, by consulting with a third reviewer (N.K.). Adapted from the Cochrane RoB tool, it includes 10 criteria covering selection bias, performance bias, detection bias, attrition bias, reporting bias, and other potential sources of biases. Each criterion was assessed and categorized as “yes,” “no,” or “unclear.” To visualize the RoB assessment, the robvis web application was used to generate traffic light plots representing domain-level judgments for each result.

### Statistical analysis

Meta-analysis was conducted if an outcome was reported in at least three studies, using R (version 4.4.1; R Foundation for Statistical Computing, Vienna, Austria) within RStudio (version 2024.12.0; Posit, Boston, MA), to estimate the overall effect using the restricted maximum likelihood method [18, 19]. The outcome variables were compared between the data obtained after the curcumin intervention and the control group. A random-effects meta-analysis was performed to synthesize the data by pooling the outcomes of the included studies. When mean and standard deviation (SD) values were unavailable, alternative metrics, such as the median and interquartile range (IQR), were converted for each outcome. If SD values were missing, they were either imputed or derived from standard error (SE) or confidence interval (CI) by the RevMan calculator, when applicable. The effect estimates were presented as standardized mean difference (SMD) with 95% CI, with *p* < 0.05 considered statistically significant. Forest and funnel plots were generated using the “meta” package (version 8.0–2) in RStudio to visualize effect estimates and assess publication bias. Egger’s test was applied to assess funnel plot asymmetry when at least three studies were included. Between-study heterogeneity was evaluated using Cochran’s Q test and the I² statistic.

A meta-regression analysis was conducted to assess the impact of curcumin dosage on upper GI-related outcomes when at least ten studies were included in the meta-analysis. Due to the high heterogeneity in study methodologies, a subgroup analysis was performed to identify potential sources of variation by categorizing studies based on curcumin dosages, intervention approaches, routes of administration, and induction methods. Curcumin dosages were classified as > 18.5 mg/kg/day or ≤ 18.5 mg/kg/day, derived from the human ADI of up to 3 mg/kg/day, calculated on the basis of a reference human body weight of 60 kg and subsequently converted to animal equivalents. If a study included several dosages, the number of animals in the control group was divided proportionally across the dosage groups. Intervention approaches were classified as protection (administration of curcumin before disease induction) and treatment (administration after disease induction). The routes of administration were categorized as oral (per oral or intragastric), intraperitoneal, and intraduodenal. Disease induction methods were categorized as administration of non-steroidal anti-inflammatory drugs (NSAIDs), ethanol exposure, stress including cold restraint stress (CRS) and water-immersion restraint stress (WRS), pyloric ligation, and other approaches such as administration of serotonin, reserpine, or combination protocols. Furthermore, when fewer than three studies were available or outcomes could not be pooled, only qualitative synthesis was performed, with findings reported through descriptive summaries of the outcomes within each category.

## Results

### Study selection

**Fig. 1:**
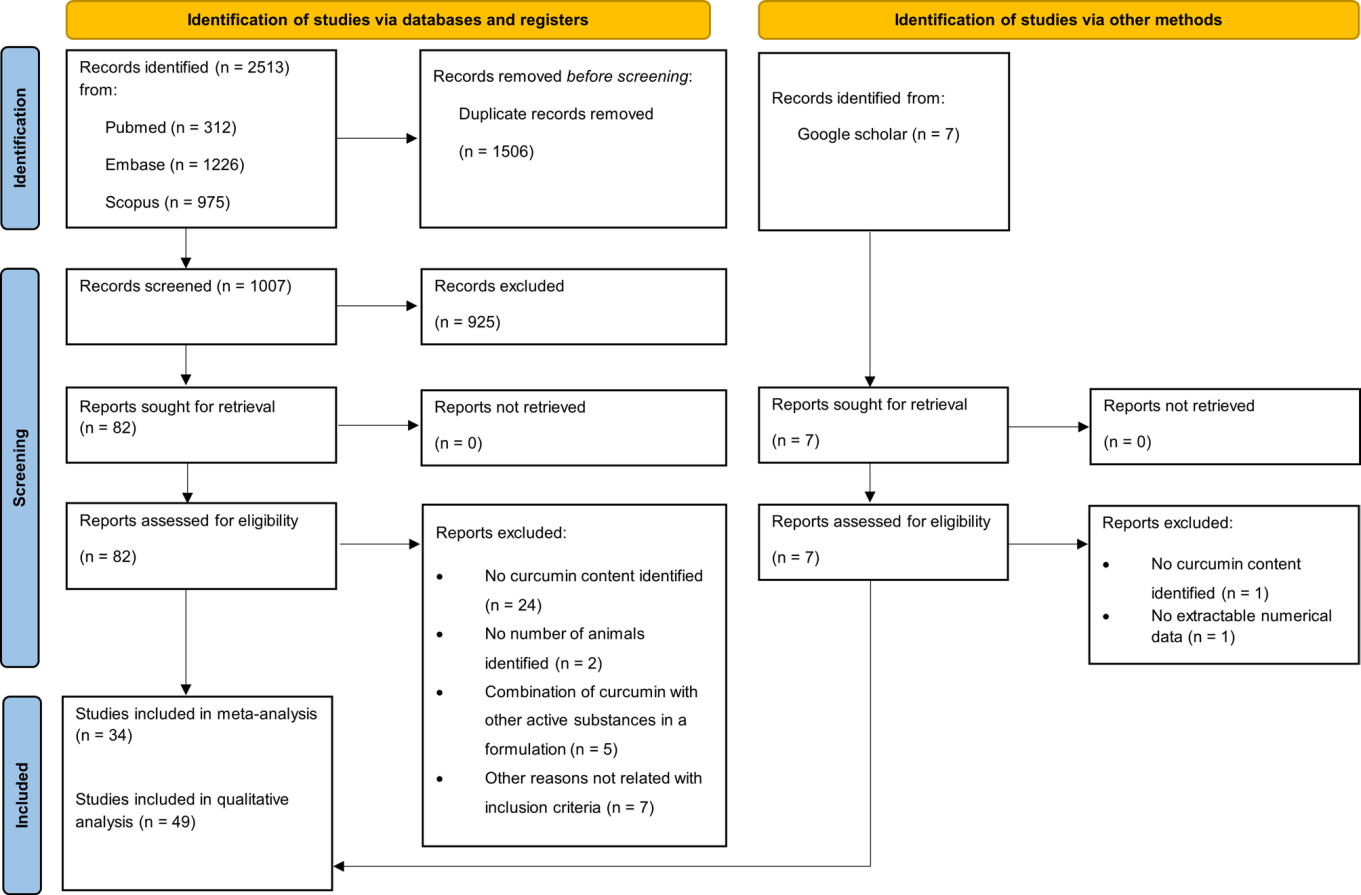
Preferred Reporting Items for Systematic Reviews and Meta-Analyses (PRISMA) flow diagram

The database search initially identified 2513 records. After removing duplicates, 1007 records remained for screening. Following title and abstract screening, 82 full-text articles were assessed for eligibility. Ultimately, 44 studies retrieved from the databases met the eligibility criteria. An additional 7 studies were identified through a Google Scholar hand search, of which 2 were excluded, resulting in 5 eligible studies. In total, 49 studies were included in the analysis (Fig.1).

### Study characteristics

A total of 49 studies were published between 2004 and 2024, of which 49 were included in the qualitative synthesis and 34 in the meta-analysis. The qualitative synthesis examined curcumin’s effects on various upper GI disorders, including gastric ulcers, diabetic gastroparesis, esophageal diseases, *H. pylori* infection, and gastritis, while the meta-analysis focused on its effects on gastric ulcers and diabetic gastroparesis in rodent models (rats and mice). The study characteristics in the meta-analysis are summarized in Table 2.

**Table 2:** Characteristics of the studies included in the meta-analysis

First author (year)	Animal species	Number/group	Induction methods	Intervention	Control	Route of administration	Frequency	Treatment duration	Intervention approach
**Gastric ulcer model**									
Swarnakar (2004)[20]	SD Rats	12	Indomethacin	Cur 10,20,40 mg/kg	Vehicle	i.p.	N/A	4.5 h	Protection
				Cur 10,20,40,60 mg/kg	Vehicle	p.o.	N/A	4.5 h	Protection
Chattopadhyay (2006)[21]	SD Rats	10	Indomethacin	Cur 5,10,25,50 mg/kg	Vehicle	i.p.	Single	4.5 h	Protection
			Pylorus ligation	Cur 5,10,25,50 mg/kg	Vehicle	i.p.	Single	4.5 h	Protection
			CRS	Cur 5,10,25 mg/kg	Vehicle	i.p.	Single	4.5 h	Protection
			50% Ethanol	Cur 10,25,50 mg/kg	Vehicle	i.p.	Single	3 h	Protection
Mahattanadul (2006)[22]	Wistar rats	6–10	Topical acetic acid	Cur 5,10,20,40,80 mg/kg	1% CMC	p.o.	b.i.d.	10 days	Protection
			80% Ethanol	Cur 5,10,20,40 mg/kg	1% CMC	p.o.	Single	1.5 h	Protection
			Repeated treatment with serotonin	Cur 20,80 mg/kg	1% CMC	p.o.	o.d.	4 days	Protection
			Pylorus ligation	Cur 5,10,20,40 mg/kg	1% CMC	i.d.	Single	4 h	Treatment
Mahattanadul (2009)[23]	Wistar rats	10	Topical acetic acid	Cur 20,40,80 mg/kg	1% CMC	p.o.	b.i.d.	10 days	Treatment
			Pylorus ligation	Cur 5,10, 20,40,80 mg/kg	1% CMC	i.d.	Single	4 h	Treatment
Mei (2009) [24]	SD rats	10	Pylorus ligation	Cur SDs (eqv. Cur 24 mg/kg)	PVP	p.o.	N/A	7 days	Protection
Tuorkey (2009)[25]	Wistar albino rats	10	Pylorus ligation	Cur 20,40,80 mg/kg	Saline	p.o.	N/A	19 h	Treatment
Mei (2011)[26]	SD rats	10	CRS	Cur SDs (eqv. Cur 24 mg/kg)	PVP	p.o.	N/A	7 days	Protection
Gandhi (2012)[27]	Wistar albino rats	6	Indomethacin	Cur 100 mg/kg	0.1% Tween 80 solution	p.o.	Single	4.5 h	Protection
Mei (2012)[28]	SD rats	10	75% Ethanol	Cur SDs (eqv. Cur 24 mg/kg)	PVP	p.o.	N/A	7 days	Protection
Thong-Ngam (2012)[29]	SD rats	6	Indomethacin	Cur 200 mg/kg	Olive oil	p.o.	N/A	8.5 h	Protection
Mei (2013)[30]	SD rats	10	Topical acetic acid	Cur SDs (eqv. Cur 24 mg/kg)	PVP	p.o.	N/A	10 days	Protection
Morsy (2013)[31]	Wistar rats	10	Combination of pylorus ligation and indomethacin	Cur 50 mg/kg	0.5% CMC	p.o.	N/A	3.5 h	Protection
Díaz-Triste (2014)[32]	Wistar rats	7	Indomethacin	Cur 3,10,30,100,300 mg/kg	CMC	p.o.	Single	3.5 h	Protection
He (2014) [33]	SD rats	10	WRS	Cur 20 mg/kg	Normal saline	p.o.	N/A	7 days	Protection
Kerdsakundee (2015)[34]	Wistar rats	8	Topical acetic acid	Cur 40 mg/kg	1% CMC	p.o.	b.i.d.	10 days	Treatment
				Cur SDs 20, 40 mg/kg	1% CMC	p.o.	o.d.	10 days	Treatment
Czekaj (2016)[35]	Wistar rats	7	WRS	Cur 2.5,10,25,50,100 mg/kg	Saline	i.g.	Single	4 h	Protection
			Pentagastrin	Cur 50 mg/kg	Saline	i.g.	Single	120 min	Treatment
			Histamine	Cur 50 mg/kg	Saline	i.g.	Single	120 min	Treatment
Kim (2016)[36]	SD rats	8	Naproxen	Cur 10,50,100 mg/kg	Vehicle	p.o.	NA	3 days	Protection
Long (2016)[37]	SD rats	8	Reserpine	Cur 100,200 mg/kg	Saline	i.g.	N/A	14 days	Treatment
Singh (2017)[38]	Wistar rats	9	Diclofenac sodium	Cur 25,50,100 mg/kg	Vehicle	p.o.	b.i.d.	10 days	Protection
Czekaj (2017)[39]	Wistar rats	8	75% Ethanol	Cur 2.5,10,50,100 mg/kg	Saline	i.g.	Single	1.5 h	Protection
Ibrahim (2019)[40]	Albino rats	9	Piroxicam	Cur 200 mg/kg	N/A	p.o.	o.d.	21 days	Treatment
Sharma (2019)[41]	Wistar rats	6	Absolute ethanol	Cur 50 mg/kg Cur LS 25,50 mg/kg	0.1% w/v CMC	p.o.	N/A	4 h	Protection
Bao (2021)[42]	C57BL/6 mice	8	Absolute ethanol	Cur 100 mg/kg	Normal saline	p.o.	Single	1 h	Treatment
Kuadkaew (2021)[43]	Wistar rats	6	Indomethacin	Cur 20 mg/kg	0.1 M Acetic acid	p.o.	o.d.	2 days	Treatment
Mahattanadul (2020) [44]	Wistar rats	6	Topical acetic acid	Cur 40 mg/kg	1% CMC	p.o.	b.i.d.	10 days	Treatment
				Cur in SMEDDS 40 mg/kg	1% CMC	p.o.	o.d.	10 days	Treatment
Ali (2022)[45]	SD rats	6	CRS	Cur 100 mg/kg and Cur in NPs 100 mg/kg	Deionized water	p.o.	N/A	14 days	Protection
Gao (2022)[46]	C57BL/6 mice	10	Cisplatin	Cur 25 mg/kg	Normal saline	i.p.	N/A	6 days	Treatment
Joshi (2023) [47]	SD rats	6	Absolute ethanol	Cur 100 mg/kg	N/A	p.o.	N/A	5 days	Protection
Al-kawaz (2024) [48]	Rats	5	Absolute ethanol	Cur 40 mg/kg	N/A	p.o.	o.d.	5 days	Treatment
Ansari (2024)[49]	Wistar rats	6	Aspirin	Cur 40 mg/kg	Corn oil	p.o.	N/A	10 days	Protection
Keçeci (2024) [50]	Wistar albino rats	8	Absolute ethanol	Cur 100 mg/kg	Corn oil	p.o.	Single	1.5 h	Protection
**Diabetic gastroparesis model**									
Jin (2013) [51]	SD rats	10	STZ	Cur 150 mg/kg	0.5% CMC-Na solution	i.g.	o.d.	6 weeks	Treatment
Xu (2013)[52]	Wistar rats	8	STZ	Cur 100,200,400 mg/kg	1% CMC-Na solution	i.g.	N/A	28 days	Treatment
Sampath (2021) [53]	Nrf2 KO and WT mice	4	HFD	Cur 200 mg/kg	N/A	p.o.	N/A	10 weeks	Treatment

b.i.d. = twice a day, *CMC* carboxymethyl cellulose, *CRS* cold restraint stress, *Cur* curcumin, *eqv.* equivalent, *i.d*. intraduodenal, *i.g.* intragastric, *i.p*. intraperitoneal, *kg* kilogram, *LS* liquisolid, *M* molar, *mg* milligram, N/A = not available, *Na* sodium,* NPs* nanoparticles, *Nrf2 KO* homozygous *Nfe2l2*^−/−^, o.d. = once a day, p.o. = orally, *PVP* polyvinylpyrrolidone, *SD* Sprague Dawley, *SDs* solid dispersions, *SMEDDS* Self-microemulsifying drug delivery systems, *STZ* streptozotocin, *WRS * water-immersion restraint stress, and *WT* wild-type

### Risk of bias in studies

Overall, an unclear RoB was observed across most of the studies, with five studies showing a high RoB (Supplementary Fig. 1). Most studies lacked adequate descriptions of the allocation sequence, allocation concealment, baseline characteristics, and failed to adjust for potential confounders, raising concerns about selection bias and affecting internal validity. Additionally, all studies lacked sufficient details on random animal housing, blinding of caregivers and/or investigators and random outcome assessment, resulting in unclear risks of performance and detection biases. For attrition bias, certain studies that reported complete outcome data were judged to be at low risk, whereas the majority provided insufficient information and were therefore rated as having an unclear risk. Moreover, four studies were classified as high risk in this domain owing to the absence of appropriate methods for handling dropouts. However, among the 34 studies included, 32 were evaluated as having a low risk of reporting bias, as their reported outcomes were consistent with those prespecified in the Methods section. Other potential sources of biases, such as industry sponsorship or funding-related conflicts of interest, were not sufficiently reported in most studies, leading to an overall unclear assessment of bias risk. Therefore, the majority of the studies (29 of 34) were assessed as having an overall unclear risk of bias due to insufficient detail, whereas five studies were judged to have a high risk of bias. 

### Effects of curcumin on upper GI-related outcomes in animal models

A summary of the main findings is presented in Table 3. Overall, curcumin demonstrated beneficial effects across animal models of upper GI disorders, including gastric ulcers, diabetic gastroparesis, esophageal diseases, *H. pylori* infection, and gastritis, as detailed below.

**Table 3:** Summary of findings on the effects of curcumin in animal models of upper gastrointestinal disorders compared with controls

Disease model	Effect of curcumin in animal models
**Gastric ulcer**	
Biochemical markers	↑ CAT, ↑ GSH, ↑ SOD, ↓ MDA, ↓ iNOS, ↓ TNF-α
Gastric acid parameters	↓ Acid output, ↓ Gastric acid volume, ↔ Gastrin, ↓ H^+^/K^+^ ATPase, ↑ pH of gastric juice, ↓ Total acidity
Ulcer parameters	↓ Ulcer index, ↓ Ulcer area, ↑ Mucosal healing index, ↑ Ulcer healing index
** Diabetic gastroparesis**	↓ Blood glucose, ↑ Gastric emptying rate
** Other GI disorders**	↓ Esophageal inflammation, ↓ *H. pylori* colonization, ↓ Gastritis severity and restored gastric tissues

*NOTE* ↑ = increase; ↓ = decrease; ↔ = no significant change

*Abbreviations*: *CAT* catalase, *GI* gastrointestinal, GSH glutathione, *H. pylori* *Helicobacter pylori*, *H*^+^/K^+^ ATPase hydrogen/potassium adenosine triphosphatase, iNOS inducible nitric oxide synthase, *MDA* malondialdehyde, *pH* potential of hydrogen,* SOD* superoxide dismutase,* TNF-α *tumor necrosis factor-alpha

### Effects of curcumin on gastric ulcers 

The effect of curcumin on gastric ulcers has been investigated in 31 studies, mainly in rats, with only two involving mice [20–50]. Sample sizes ranged from 5 to 12 animals per group. Various methods were used to induce gastric ulcers, such as ethanol (9 studies), indomethacin (6 studies), pylorus ligation (5 studies), topical acetic acid (5 studies), cold restraint stress (3 studies), and water-immersion restraint stress (2 studies), among others. The curcumin dosages tested ranged from 2.5 to 300 mg/kg/day, with the majority administered orally. Three studies administered curcumin via the intraperitoneal route, and two via the intraduodenal route. Intervention durations varied from 1 hour to 21 days. The most common curcumin intervention approach was protection, reported in 19 studies; treatment was reported in 10 studies; and both approaches were reported in 2 studies.

**Fig. 2:**
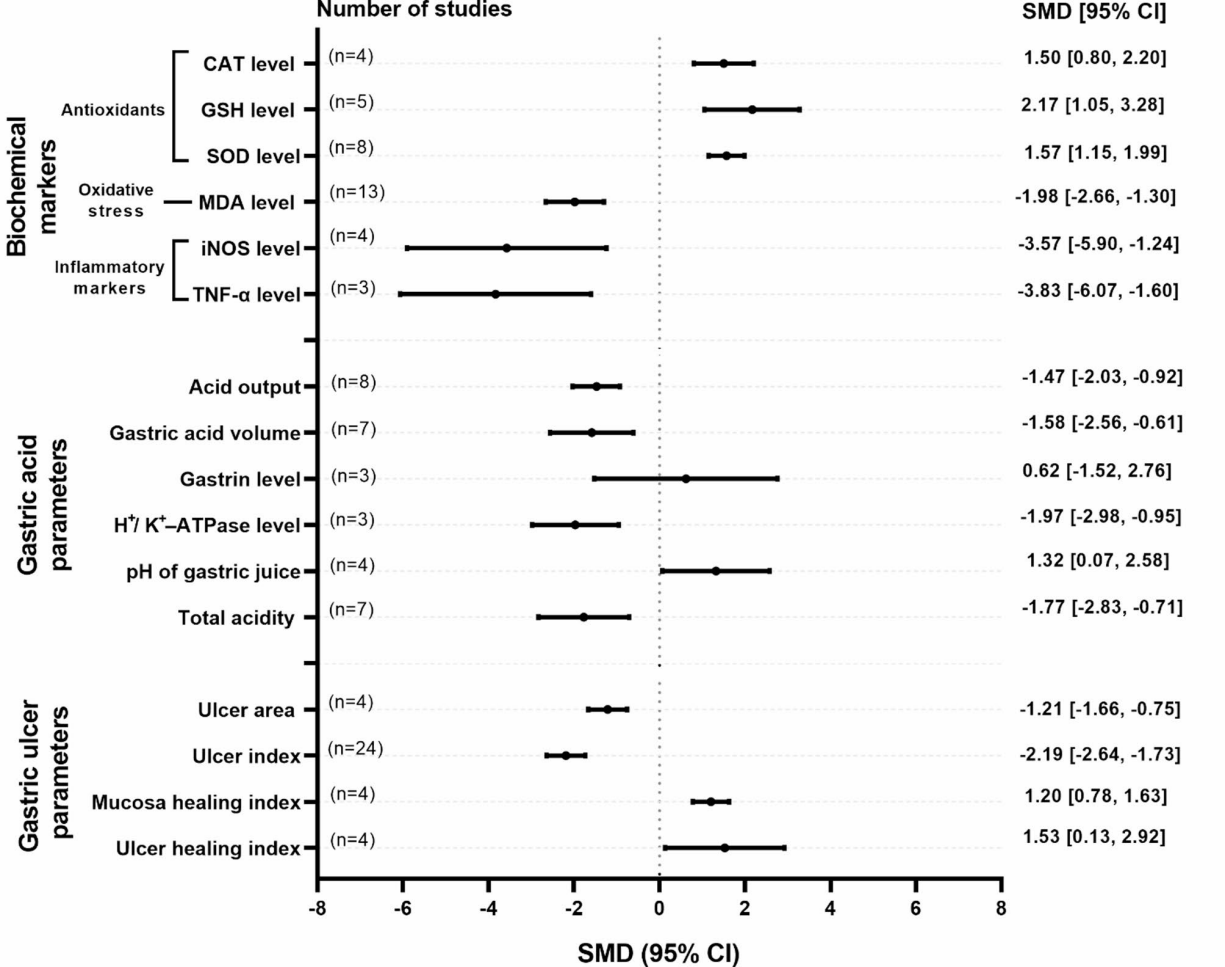
Forest plot summary of the standardized mean difference (SMD) in biochemical markers, gastric acid parameters, and gastric ulcer parameters following curcumin intervention versus control in gastric ulcer-induced animals

Curcumin administration significantly improved biochemical markers, gastric acid parameters, and gastric ulcer parameters compared with the control in ulcerated animals (Fig. 2). Specifically, curcumin significantly increased antioxidants, including CAT (SMD = 1.50; 95% CI: 0.80–2.20; *p* < 0.0001), SOD (SMD = 1.57; 95% CI: 1.15–1.99; *p* < 0.0001), and GSH levels (SMD = 2.17; 95% CI: 1.05–3.28; *p* = 0.0001). Concurrently, curcumin significantly reduced oxidative stress marker, as evidenced by a decrease in MDA levels (SMD = −1.98; 95% CI: −2.66–−1.30; *p* < 0.0001). Additionally, it also significantly decreased inflammatory markers, including iNOS (SMD = −3.57; 95% CI: −5.90–−1.24; *p* = 0.0027) and TNF-α levels (SMD = −3.83; 95% CI: −6.07–−1.60; *p* = 0.0008). Furthermore, curcumin significantly affected gastric acid parameters by reducing acid output (SMD = −1.47; 95% CI: −2.03–−0.92; *p* < 0.0001), gastric acid volume (SMD = −1.58; 95% CI: −2.56–−0.61; *p* = 0.0015), H^+^/K^+^-ATPase levels (SMD = −1.97; 95% CI: −2.98–−0.95; *p* = 0.0001), and total acidity (SMD = −1.77; 95% CI: −2.83–−0.71; *p* = 0.0011). Simultaneously, it significantly increased the pH of gastric acid (SMD = 1.32; 95% CI: 0.07–2.58; *p* = 0.0386). However, curcumin intervention did not significantly impact gastrin levels (SMD = 0.62; 95% CI: −1.52–2.76; *p* = 0.5690). In terms of gastric ulcer parameters, curcumin significantly reduced the ulcer area (SMD = −1.21; 95% CI: −1.66–−0.75; *p* < 0.0001) and ulcer index (SMD = −2.19; 95% CI: −2.64–−1.73; *p* < 0.0001), while notably increasing the mucosal healing (SMD = 1.20; 95% CI: 0.78–1.63; *p* < 0.0001) and ulcer healing indices (SMD = 1.53; 95% CI: 0.13–2.92; *p* = 0.0318). However, most analyses demonstrated substantial heterogeneity (I² >50%) across the outcomes, except for CAT levels, SOD levels, H^+^/K^+^-ATPase levels, mucosal healing index, and ulcer area. Detailed information is provided in Supplementary Figs. 2–16. 

### Effects of curcumin on diabetic gastroparesis disease

**Fig. 3:**
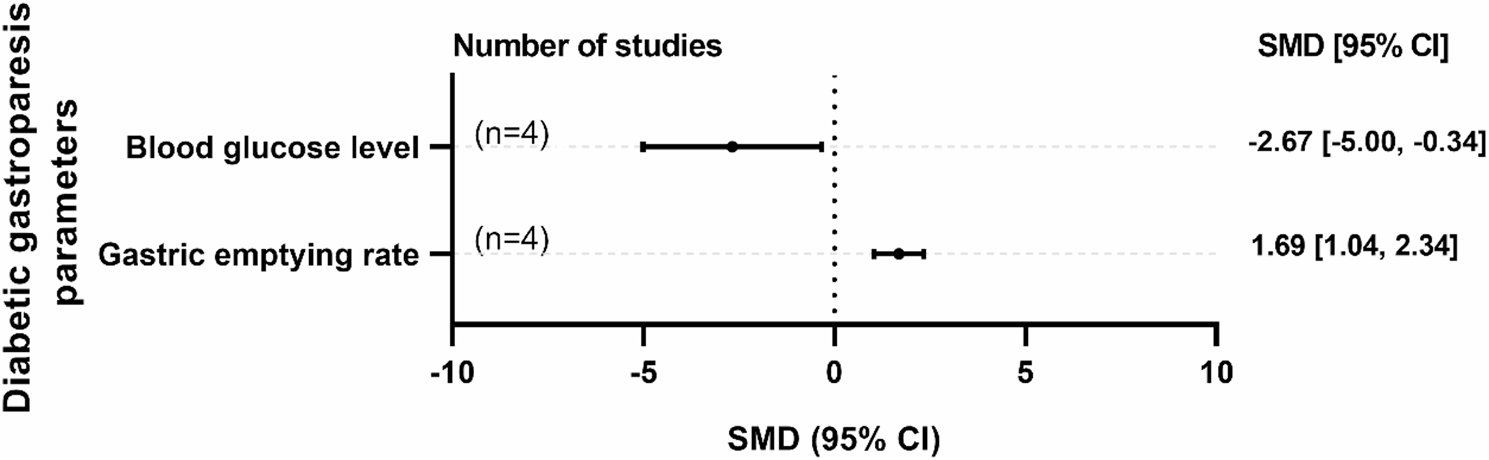
Forest plot summary of the standardized mean difference (SMD) in blood glucose levels and gastric emptying rate following curcumin intervention versus control in diabetic gastroparesis-induced animals

Three studies assessed curcumin’s effects on diabetic gastroparesis in rodent models (mice and rats), with group sizes ranging from 4 to 10 [51–53]. The induction methods included streptozotocin in two studies and a high-fat diet in one study. The curcumin dosages ranged from 100 to 400 mg/kg/day, administered either intragastrically (in 2 studies) or orally (in 1 study). The intervention periods ranged from 4 to 10 weeks, with all studies focusing on a treatment approach. Curcumin administration significantly improved diabetic gastroparesis-related outcomes compared with the control in animal models (Fig. 3). It effectively reduced blood glucose levels (SMD = −2.67; 95% CI: −5.00–−0.34; *p* = 0.0248) and enhanced the gastric emptying rate (SMD = 1.69; 95% CI: 1.04–2.34; *p *< 0.0001). Nevertheless, the analysis revealed high heterogeneity (I² >50) in blood glucose levels but not in the gastric emptying rate. Detailed information is provided in Supplementary Figs. 17–18. Due to the high heterogeneity of most outcomes, subgroup analyses were performed.

#### Subgroup analyses

Despite performing subgroup analyses based on curcumin dosages, intervention approaches, routes of administration, and induction methods, high heterogeneity remained in most cases (Supplementary Tables 2–5). Across these comparisons, most outcomes remained statistically significance. The heterogeneity was reduced (I² < 50%) in certain subgroups compared with the overall analysis. Specifically, lower heterogeneity was observed for SOD levels at doses > 18.5 mg/kg/day and in the protective approach; MDA levels in the treatment approach; acid output at doses ≤ 18.5 mg/kg/day, in the treatment approach, and with oral administration; gastric acid volume in the treatment approach and pyloric ligation models; ulcer index in ethanol-induced models; and the mucosal healing index at doses > 18.5 mg/kg/day. With respect to dosages, curcumin consistently produced significant effects at doses > 18.5 mg/kg/day. At doses ≤ 18.5 mg/kg/day, significant effects were also observed for acid output and ulcer index, but not for ulcer area. Regarding the intervention approaches, both protective and treatment strategies produced significant effects on MDA levels, acid output, gastric acid volume, and ulcer index. In contrast, the protective strategy showed no significant impact on gastric juice pH or total acidity. For the routes of administration, most outcomes were significant in the oral subgroup, except for the ulcer healing index. Moreover, the ulcer index was significantly affected by both the oral and intraperitoneal routes. Across induction methods, curcumin exerted the effects on most outcomes, indicating consistent effects regardless of the ulcer induction model applied.

#### Meta-regression analyses

**Fig. 4:**
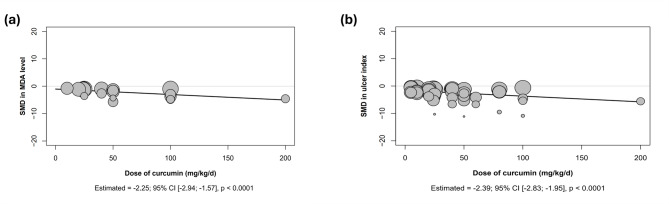
Meta-regression of curcumin dosage on (**a**) malondialdehyde (MDA) levels and (**b**) ulcer index in gastric ulcer-induced animals

Meta-regression analyses were performed for two outcomes, MDA levels and ulcer index. The findings demonstrated significant correlations between curcumin dosage and MDA levels, as well as between curcumin dosage and the ulcer index in gastric ulcer-induced animals, as shown in Fig. 4. This indicated that higher dosages results in lower MDA levels and a reduced ulcer index.

### Effects of curcumin on esophageal diseases

Four animal studies investigated the effects of curcumin (3.8–200 mg/kg for 24 hours to 30 days) on esophageal diseases, as shown in Supplementary Fig. 20 [54–57]. Curcumin exhibited protective and reparative effects on esophageal tissue in animal models of esophagitis by restoring the muscularis mucosa, enhancing collagen deposition, and reducing keratin layer thickness. It also significantly lowered serum aspartate aminotransferase (AST), alanine aminotransferase (ALT), and lactate dehydrogenase (LDH) levels. In gastroesophageal reflux disease (GERD) models, curcumin inhibited the development of acute acid reflux esophagitis, lowered the esophageal coefficient, reduced esophageal perforation, prevented disruption of the lamina propria, and attenuated inflammatory cell infiltration compared with the control; however, it did not completely prevent tissue damage.

### Effects of curcumin on *H. pyroli* infection

Seven animal studies investigated the effects of curcumin on *H. pylori *infection in the stomach, as summarized in Supplementary Fig. 20 [58–64]. Animals were infected with *H. pylori* strains either isolated from patients or obtained from the American Type Culture Collection (mainly SS1 or AM strains). Infection was confirmed through urease tests and histopathological examination. Curcumin (25–600 mg/kg/day), with one study reporting an absolute dose of 20 µg/day, was administered in various formulations, including suspension, nanoemulsion, and lipidic solution, for durations ranging from 7 days to 27 weeks post-infection. The studies found that curcumin significantly decreased *H. pylori* colonization and inflammation markers in the stomach tissues of infected animals, including the activities of matrix metalloproteinase (MMP)−3 and MMP-9, as well as pro-inflammatory molecules such as activator protein-1 and cyclooxygenase (COX)−2. Simultaneously, curcumin increased the levels of peroxisome proliferator-activated receptor gamma (PPAR-γ), and inhibitor of kappa B alpha(IκB-α). Histopathological analysis showed that curcumin treatment restored epithelial integrity, reduced neutrophil infiltration and pit cell inflammation, prevented epithelial necrosis, decreased nuclear factor-kappa B (NF-κB) p65 immunoreactivity and macromolecular leakage, as well as preserved the gastric mucosal structure in infected gastric tissues. Additionally, it lowered immunoglobulin G (IgG) against *H. pylori*, interferon-gamma (IFN-γ), and gastrin levels while increasing interleukin (IL)−4 levels in serum, suggesting a role in balancing the immune system and the response to *H. pylori* infection.

### Effects of curcumin on gastritis

Four animal studies investigated the effects of curcumin on gastritis, as demonstrated in Supplementary Fig. 20 [65–68]. Curcumin (30–500 mg/kg) was administered for 14 days to 18 weeks. Across studies, histopathological findings indicated that curcumin treatment reduced gastric mucosal injury, restored vascular integrity, promoted capillary sprouting, and attenuated neutrophil infiltration in gastric tissues. It also reduced MDA levels, suppressed myeloperoxidase and COX-2 activities, downregulated the expression of NF-κB p65, TNF-α, IL-6, and IL-1β, and diminished DNA fragmentation, while enhancing CAT, GSH, and SOD activities in gastritis-induced animals. Additionally, gene expression analysis revealed the downregulation of inflammatory markers, including pro-inflammatory cytokines, chemokines, and toll-like receptors in the curcumin group. Biochemical analyses further demonstrated reductions in serum nitric oxide and sialic acid, along with an increase in total antioxidant capacity. Another study reported that curcumin prevented gastric inflammation, as evidenced by the absence of neutrophil infiltration, lack of atrophy, and preserved glandular morphology.

## Discussion

This systematic review and meta-analysis highlights the potential therapeutic benefits of curcumin for upper GI diseases, reinforcing its relevance in gastroenterology research. Overall, the evidence suggests that curcumin exerts beneficial effects across multiple upper GI disorders, including gastric ulcers, diabetic gastroparesis, esophageal diseases, *H. pylori* infection, and gastritis. By synthesizing evidence from gastric ulcer-induced animal studies, this research demonstrates curcumin’s ability to improve key biochemical markers, including CAT, GSH, SOD, MDA, iNOS, and TNF-α, which are critical in oxidative stress and inflammatory responses. Additionally, curcumin could modulate gastric acid parameters by reducing acid output, gastric acid volume, H^+^/K^+^-ATPase levels, and total acidity while increasing gastric acid pH, suggesting its favorable effects against acid-related disorders. Its ability to reduce ulcer area and ulcer index while promoting mucosal and ulcer healing indices further supports its therapeutic potential. Moreover, curcumin improves diabetic gastroparesis outcomes by lowering blood glucose levels and enhancing the gastric emptying rate. These findings provide a foundation for further investigation into curcumin’s clinical applications, emphasizing the need for well-designed human studies to validate its efficacy and safety in treating upper GI diseases.

Curcumin may help mitigate gastric ulcers in animals by reducing aggressive factors (e.g. oxidative stress, inflammation, and gastric hyperacidity), whereas enhancing protective factors (e.g. antioxidant defenses, ulcer healing, and mucosal repair). It can be reasoned that curcumin possesses pleiotropic properties, including antioxidant, anti-inflammatory, and tissue-repairing actions, all of which contribute to its therapeutic effects [10]. This finding aligns with the study by Tuorkey and Karolin (2009), which reported that curcumin’s anti-ulcer effects involve reducing gastric acid hypersecretion, lipid peroxidation, pro-inflammatory cytokines, and apoptotic incidence [25]. Due to its multifaceted mechanisms of action, curcumin may offer enhanced therapeutic potential compared to agents with a single mechanism, making it a valuable adjunct to standard care in managing peptic ulcer disease. Furthermore, the effects of curcumin on MDA levels and ulcer index may be dose dependent. This may be attributed to curcumin at lower doses not reaching sufficient concentrations to effectively exert biological activities, likely due to its physicochemical and pharmacokinetic properties [69]. Curcumin is well known for its low systemic bioavailability, which results from poor solubility, limited absorption, low intrinsic activity, rapid metabolism, and fast elimination from the body [70, 71]. Accordingly, optimizing the dosage of curcumin is essential to balance therapeutic benefits with safety considerations.

Based on subgroup analyses, curcumin at doses exceeding 18.5 mg/kg/day—calculated with reference to the human ADI of 3 mg/kg/day—produced significant effects across most upper GI outcomes. While at doses ≤ 18.5 mg/kg/day, significant effects were observed for acid output and ulcer index, but not for ulcer area. This may be explained by the fact that the ulcer index, which integrates both lesion number and severity relative to the mucosal surface, provides a more sensitive and reproducible measure of gastric injury than the ulcer area, which represents an absolute measurement [72]. With respect to intervention approaches, both protective and treatment strategies significantly influenced outcomes. However, the protective approach did not demonstrate significant effects on pH of gastric juice and total acidity. This may be attributed to curcumin’s ability to downregulate H⁺/K⁺-ATPase, the proton pump responsible for gastric acid secretion, particularly under conditions of acid hypersecretion following ulcer induction [33]. In addition, the oral route significantly affected most outcomes, except for the ulcer healing index. This finding may reflect that orally administered curcumin could lead to insufficient systemic exposure to support the prolonged and complex repair processes required for ulcer healing, such as angiogenesis and collagen deposition [25, 73]. Furthermore, curcumin exerted consistent benefits across all ulcer induction models, suggesting its broad therapeutic potential against diverse ulcerogenic factors.

Curcumin also improved diabetic gastroparesis–related outcomes in animals by reducing elevated blood glucose levels, which are known to aggravate gastric motility disturbances in diabetic conditions. It further enhanced the gastric emptying rate, indicating its potential to alleviate delayed gastric motility. Several possible mechanisms underlying the effects of curcumin have been reported, with antioxidant and anti-inflammatory effects being the most prominent. Curcumin promotes pancreatic islet cell survival by reducing reactive oxygen species formation, suppressing pro-inflammatory cytokine activation, and inhibiting endoplasmic reticulum-mediated, mitochondria-dependent, and mitochondria-independent apoptotic pathways [74]. It also enhances the expression of AMP-activated protein kinase and PPAR-γ, reduces NF-κB proteins, and upregulates the expression of stem cell factor/c-kit, key components involved in coordinating gastric muscular contractions in diabetic mice [51, 74]. These findings suggest that curcumin may offer dual benefits in managing diabetic gastroparesis by addressing both the underlying hyperglycemia and impaired gastric motility.

Curcumin may offer beneficial effects against esophageal disorders, *H. pylori *infection, and gastritis. These findings are consistent with previous studies, which reported that curcumin suppressed the expression of IL-6 and IL-8 in an acid-induced esophageal epithelial cell line, with the NF-κB pathway potentially playing a key role [75]. *H. pylori *is recognized as a primary human pathogen associated with chronic gastritis, peptic ulcer disease, and an elevated risk of gastric adenocarcinoma [76]. Curcumin counteracted *H. pylori *pathogenicity by suppressing virulence factor–induced inflammation and oxidative stress that damage the gastric mucosa [77]. This result is supported by Judaki et al. (2017), who reported that the addition of curcumin (700 mg, taken orally three times daily for 4 weeks) to triple therapy regimes significantly improved the *H. pylori *eradication rate and reduced active, chronic, and endoscopic inflammation scores in 50 chronic gastritis patients, compared to baseline and triple therapy alone [78].

This systematic review and meta-analysis represents the first comprehensive and up-to-date evaluation of curcumin’s effects on various upper GI disorders in animals, highlighting its potential across several key areas. One of the study’s strengths is its systematic approach to evaluating a broad range of upper GI diseases and conditions, offering a thorough overview of the topic. Additionally, it enhances comparability by standardizing curcumin doses across various formulations, thereby providing a clearer understanding of the effective doses required to achieve therapeutic effects. The use of advanced data extraction techniques, such as WebPlotDigitizer, allowed for more thorough data collection.

Although this study provides valuable insights into the effects of curcumin on various upper GI diseases and conditions in animals, there are several limitations to consider. First, the limited availability of studies on certain outcomes, such as gastric blood flow, pepsin levels, mucosa regeneration index, and prostaglandin E2 levels, hindered the feasibility of pooling effect estimates for those outcomes. Second, high heterogeneity was observed across several analyses, likely influenced by variation in curcumin dosages, formulation types, animal species, induction methods, the absence of standardized GI assessments, and other variables. Despite efforts through subgroup analyses, the sources of heterogeneity could not be precisely identified. In addition, imputing missing data and extracting values using WebPlotDigitizer may not have yielded the exact values as in the original study data, which may have affected the results. Furthermore, the possibility of publication bias cannot be excluded, as studies with negative or null results may be underrepresented in the published literature. Third, most animal studies did not provide sufficient information to assess the RoB, resulting in a classification of “unclear” RoB. Additionally, certain upper GI diseases, such as dyspepsia, cannot be effectively assessed in animal models, as these evaluations often rely on subjective symptom reporting, which animals are unable to provide. However, curcumin—with its diverse bioactivities—may offer therapeutic potential for managing functional dyspepsia, a condition characterized by complex pathology, through the modulation of gut inflammation [79]. Lastly, the findings from animal studies cannot be directly extrapolated to humans. Biological differences between species, such as variations in metabolic rates, immune responses, and receptor expressions, can lead to differences in the observed effects. These intrinsic variations necessitate cautious interpretation of the results. Further clinical research is needed to verify the therapeutic efficacy of curcumin on upper GI diseases and to validate its translational applicability in humans.

## Conclusion

This systematic review and meta-analysis suggests potential benefits of curcumin in animal models of upper GI disorders, including gastric ulcers, diabetic gastroparesis, esophageal diseases, *H. pylori* infection, and gastritis. Curcumin was associated with improvements in upper GI-related outcomes, such as biochemical markers, gastric acid parameters, gastric ulcer parameters, and gastric emptying rate. Additionally, its impact on MDA levels and gastric ulcer index may be dose-dependent. Further clinical investigations are needed to confirm these findings and to validate its translational applicability in humans.

## Supplementary Information

Supplementary Material 1

## Data Availability

The datasets used and/or analysed during the current study are available from the corresponding author on reasonable request.

## Abbreviations

ALT: Alanine aminotransferase
AST: Aspartate aminotransferase
CAT: Catalase
CI: Confidence interval
GERD: Gastroesophageal reflux disease
GI: Gastrointestinal
GSH: Glutathione
H⁺/K⁺ ATPase: Hydrogen-potassium adenosine triphosphatase
*H. pylori*: *Helicobacter pylori*
IgG: Immunoglobulin G
IFN-γ: Interferon gamma
IL: Interleukin
iNOS: Inducible nitric oxide synthase
IκB-α: Inhibitor of kappa B alpha
MDA: Malondialdehyde
MMP: Matrix metalloproteinase
NF-κB: Nuclear factor kappa B
NSAIDs: Non-steroidal anti-inflammatory drugs
PPAR-γ: Peroxisome proliferator-activated receptor gamma
PRISMA-P: Preferred Reporting Items for Systematic Reviews and Meta-Analyses Protocol
PROSPERO: International prospective register of systematic reviews
RoB: Risk of bias
SD: Standard deviation
SE: Standard error
SMD: Standardized mean difference
SOD: Superoxide dismutase
TNF-α: Tumor necrosis factor-alpha
SYRCLE: Systematic Review Centre for Laboratory animal Experimentation
