# Supplementary material for: Therapeutic effects of curcumin on upper gastrointestinal diseases: a systematic review and meta-analysis of animal studies
Source: BMC Complement Med Ther. 2025 Dec 5;26:54. doi: 10.1186/s12906-025-05175-4 (PMC12892551; doi:10.1186/s12906-025-05175-4)
Supplement: Supplementary file 1 — Supplementary Material 1 [file 12906_2025_5175_MOESM1_ESM.pdf]

Supplementary Information

Therapeutic Effects of Curcumin on Upper Gastrointestinal Diseases: A Systematic Review and Meta-Analysis of Animal Studies

Kankanit Yeerong[1,2], Ratchanon Inpan[1,2,3], Jakkrit Aisara[1,2], Kornvipa Settakorn[1,2], Mingkwan Na Takuathung[1,2], Nithi Thinrungroj[4], Nut Koonrungsesomboon[1,2*]

[1] Clinical Research Center for Food and Herbal Product Trials and Development (CR-FAH), Faculty of Medicine, Chiang Mai University, Chiang Mai 50200, Thailand

[2] Department of Pharmacology, Faculty of Medicine, Chiang Mai University, Chiang Mai 50200, Thailand

[3] Office of Research Administration, Chiang Mai University, Chiang Mai 50200, Thailand

[4] Division of Gastroenterology, Department of Internal Medicine, Faculty of Medicine, Chiang Mai University, Chiang Mai 50200, Thailand

[*]**Corresponding author** Nut Koonrungsesomboon, M.D., Ph.D., Department of Pharmacology, Faculty of Medicine, Chiang Mai University, Chiang Mai 50200, Thailand; Email: nkoonrung@gmail.com and nut.koonrung@cmu.ac.th; Tel.: +66-53-935352

Supplementary Table captions

Supplementary Table 1 Search strategies

Supplementary Table 2 Standardized mean difference in biochemical markers following curcumin intervention versus control in gastric ulcer-induced animals

Supplementary Table 3 Standardized mean difference in gastric acid parameters following curcumin intervention versus control in gastric ulcer-induced animals

Supplementary Table 4 Standardized mean difference in gastric ulcer parameters following curcumin intervention versus control in gastric ulcer-induced animals

Supplementary Table 5 Standardized mean difference in diabetic gastroparesis parameters following curcumin intervention versus control in diabetic gastroparesis-induced animals

Supplementary Figure captions

Supplementary Fig. 1 Risk of bias assessment

Supplementary Fig. 2 Forest plot and funnel plot of catalase (CAT) level comparing curcumin treatment to vehicle control in gastric ulcer-induced animals

Supplementary Fig. 3 Forest plot and funnel plot of glutathione (GSH) level comparing curcumin treatment to vehicle control in gastric ulcer-induced animals

Supplementary Fig. 4 Forest plot and funnel plot of superoxide dismutase (SOD) level comparing curcumin treatment to vehicle control in gastric ulcer-induced animals

Supplementary Fig. 5 Forest plot and funnel plot of malondialdehyde (MDA) level comparing curcumin treatment to vehicle control in gastric ulcer-induced animals

Supplementary Fig. 6 Forest plot and funnel plot of inducible nitric oxide synthase (iNOS) level comparing curcumin treatment to vehicle control in gastric ulcer-induced animals

Supplementary Fig. 7 Forest plot and funnel plot of tumor necrosis factor-alpha (TNF-α) comparing curcumin treatment to vehicle control in gastric ulcer-induced animals

Supplementary Fig. 8 Forest plot and funnel plot of acid output comparing curcumin treatment to vehicle control in gastric ulcer-induced animals

Supplementary Fig. 9 Forest plot and funnel plot of gastric acid volume comparing curcumin treatment to vehicle control in gastric ulcer-induced animals

Supplementary Fig. 10 Forest plot and funnel plot of gastrin level comparing curcumin treatment to vehicle control in gastric ulcer-induced animals

Supplementary Fig. 11 Forest plot and funnel plot of hydrogen-potassium adenosine triphosphatase (H^+/K^+ ATPase) comparing curcumin treatment to vehicle control in gastric ulcer-induced animals

Supplementary Fig. 12 Forest plot and funnel plot of potential of hydrogen (pH) of gastric acid comparing curcumin treatment to vehicle control in gastric ulcer-induced animals

Supplementary Fig. 13 Forest plot and funnel plot of total acidity comparing curcumin treatment to vehicle control in gastric ulcer-induced animals

Supplementary Fig. 14 Forest plot and funnel plot of ulcer area comparing curcumin treatment to vehicle control in gastric ulcer-induced animals

Supplementary Fig. 15 Forest plot and funnel plot of ulcer index comparing curcumin treatment to vehicle control in gastric ulcer-induced animals

Supplementary Fig. 16 Forest plot and funnel plot of mucosa healing index comparing curcumin treatment to vehicle control in gastric ulcer-induced animals

Supplementary Fig. 17 Forest plot and funnel plot of ulcer healing index comparing curcumin treatment to vehicle control in gastric ulcer-induced animals

Supplementary Fig. 18 Forest plot and funnel plot of blood glucose level comparing curcumin treatment to vehicle control in gastric ulcer-induced animals

Supplementary Fig. 19 Forest plot and funnel plot of gastric emptying rate comparing curcumin treatment to vehicle control in gastric ulcer-induced animals

Supplementary Fig. 20 Summary of curcumin's effect on esophageal disorder, *H. pyroli* infection, and gastritis

Supplementary Table 1

Database	Search string
PUBMED	("Curcumin"[MeSH Terms] OR "Curcumin"[All Fields] OR "Curcuma"[All Fields] OR "Curcuminoids"[All Fields] OR "Turmeric"[MeSH Terms] OR "Turmeric"[All Fields] OR "diferuloylmethane"[All Fields]) AND ("Peptic Ulcer"[MeSH Terms] OR "Peptic Ulcer"[All Fields] OR "Peptic Ulcers"[All Fields] OR "Peptic Ulceration"[All Fields] OR "Stomach Ulcer"[All Fields] OR "Gastric Ulcer"[MeSH Terms] OR "Gastric Ulcer"[All Fields] OR "Gastric Ulcers"[All Fields] OR "Duodenal Ulcer"[MeSH Terms] OR "Duodenal Ulcer"[All Fields] OR "Duodenal Ulcers"[All Fields] OR "Gastrointestinal Ulcer"[All Fields] OR "Gastrointestinal Ulceration"[All Fields] OR "Gastritis"[MeSH Terms] OR "Gastritis"[All Fields] OR "Chronic Gastritis"[All Fields] OR "Esophagitis"[MeSH Terms] OR "Esophagitis"[All Fields] OR "Reflux Esophagitis"[All Fields] OR "Gastroesophageal Reflux"[MeSH Terms] OR "Gastroesophageal Reflux"[All Fields] OR "GERD"[All Fields] OR "Gastroesophageal Reflux Disease"[All Fields] OR "Dyspepsia"[MeSH Terms] OR "Dyspepsia"[All Fields] OR "Functional Dyspepsia"[All Fields] OR "Gastroparesis"[MeSH Terms] OR "Gastroparesis"[All Fields] OR "Barrett Esophagus"[MeSH Terms] OR "Barrett Esophagus"[All Fields] OR "Heartburn"[MeSH Terms] OR "Heartburn"[All Fields] OR "Gastrointestinal Hemorrhage"[MeSH Terms] OR "Gastrointestinal Hemorrhage"[All Fields] OR "Gastrointestinal Haemorrhage"[All Fields] OR "Gastrointestinal Bleeding"[All Fields] OR "Upper Gastrointestinal Bleeding"[All Fields] OR "Gastrointestinal Perforation"[MeSH Terms] OR "Gastrointestinal Perforation"[All Fields] OR "Digestive Perforation"[All Fields] OR "Marginal Ulcer"[All Fields] OR "Curling Ulcer"[All Fields])
EMBASE	('curcumin'/exp OR curcumin:ab,ti OR curcuma:ab,ti OR curcuminoids:ab,ti OR 'turmeric'/exp OR turmeric:ab,ti OR diferuloylmethane:ab,ti) AND ('peptic ulcer'/exp OR 'peptic ulcer':ab,ti OR 'stomach ulcer':ab,ti OR 'gastric

	ulcer'/exp OR 'gastric ulcer':ab,ti OR 'duodenal ulcer'/exp OR 'duodenal ulcer':ab,ti OR 'gastrointestinal ulcer':ab,ti OR 'gastrointestinal ulceration':ab,ti OR 'gastritis'/exp OR gastritis:ab,ti OR 'chronic gastritis':ab,ti OR 'esophagitis'/exp OR esophagitis:ab,ti OR 'reflux esophagitis':ab,ti OR 'gastroesophageal reflux'/exp OR 'gastroesophageal reflux':ab,ti OR GERD:ab,ti OR 'gastroesophageal reflux disease':ab,ti OR 'dyspepsia'/exp OR dyspepsia:ab,ti OR 'functional dyspepsia':ab,ti OR 'gastroparesis'/exp OR gastroparesis:ab,ti OR 'barrett esophagus'/exp OR 'barrett esophagus':ab,ti OR 'heartburn'/exp OR heartburn:ab,ti OR 'gastrointestinal hemorrhage'/exp OR 'gastrointestinal hemorrhage':ab,ti OR 'gastrointestinal haemorrhage':ab,ti OR 'gastrointestinal bleeding':ab,ti OR 'upper gastrointestinal bleeding':ab,ti OR 'gastrointestinal perforation'/exp OR 'gastrointestinal perforation':ab,ti OR 'digestive perforation':ab,ti OR 'marginal ulcer':ab,ti OR 'curling ulcer':ab,ti)
SCOPUS	TITLE-ABS-KEY ('peptic ulcer' OR 'peptic ulcers' OR 'peptic ulceration' OR 'peptic ulcerations' OR 'stomach ulcer' OR 'stomach ulcers' OR 'stomach ulceration' OR 'stomach ulcerations' OR 'gastric ulcer' OR 'gastric ulcers' OR 'gastric ulceration' OR 'gastric ulcerations' OR 'esophagus ulcer' OR 'esophagus ulcers' OR 'esophagus ulceration' OR 'esophagus ulcerations' OR 'peptic ulcer disease' OR 'duodenal ulcer' OR 'duodenal ulcers' OR 'duodenal ulceration' OR 'duodenal ulcerations' OR 'duodenum ulcer' OR 'duodenum ulcers' OR 'duodenum ulceration' OR 'duodenum ulcerations' OR 'gastrointestinal ulcer' OR 'gastro-intestinal ulcer' OR 'gastrointestinal ulcers' OR 'gastro-intestinal ulcers' OR 'gastrointestinal ulceration' OR 'gastro-intestinal ulceration' OR 'gastrointestinal ulcerions' OR 'gastro-intestinal ulcerions' OR 'gastric mucosa' OR 'gastritis' OR 'helicobacter pylori' OR 'gastric campylobacter-like organism' OR 'campylobacter pylori' OR 'h. pylori' OR 'gerd' OR 'gastroesophageal reflux' OR 'gastroesophageal reflux disease' OR 'reflux esophagitis' OR 'esophagitis' OR 'dyspepsia' OR 'functional dyspepsia' OR 'epigastric pain' OR 'epigastric pains' OR 'gastroparesis' OR 'hiatal hernia' OR 'esophageal stricture' OR 'esophageal strictures' OR 'barrett esophagus' OR 'barretts esophagus' OR 'chronic gastritis' OR 'duodenogastric reflux disease' OR 'duodeno-

	gastric reflux disease' OR 'heartburn' OR 'gastrointestinal hemorrhage' OR 'gastrointestinal haemorrhage' OR 'gastrointestinal-hemorrhage' OR 'gastrointestinal-haemorrhage' OR 'gastrointestinal bleed' OR 'gastro-intestinal bleed' OR 'gastrointestinal bleeding' OR 'gastro-intestinal bleeding' OR 'gastrointestinal perforation' OR 'gastro-intestinal perforation' OR 'digestive hemorrhage' OR 'digestive bleeding' OR 'digestive perforation' OR 'marginal ulcer' OR 'curling ulcer' OR 'epigastric burn' OR 'gastroesophageal reflux' OR 'gastro-esophageal reflux' OR 'gastric acid reflux' OR 'gastro-oesophageal reflux' OR 'digestive disorder' OR 'bleed ulcer' OR 'bebleed ulcer' OR 're-bleed ulcer' OR 'digestive complication' OR 'gastrointestinal complication' OR 'gastrointestinal complications' OR 'gastro-intestinal complication' OR 'gastro-intestinal complications' OR 'digestive disease' OR 'digestive diseases' OR 'digestive system diseases' OR 'esophageal diseases' OR 'esophageal disease') AND<br>TITLE-ABS-KEY ('curcumin' OR 'curcuma' OR 'curcuminoids' OR 'turmeric' OR 'diferuloylmethane')
Google scholar	("Curcumin" OR "Curcuma" OR "Curcuminoids" OR "turmeric" OR "diferuloylmethane")<br>("peptic ulcer" OR "peptic ulcers" OR "peptic ulceration" OR "peptic ulcerations" OR<br>"stomach ulcer" OR "stomach ulcers" OR "stomach ulceration" OR "stomach ulcerations" OR<br>"gastric ulcer" OR "gastric ulcers" OR "gastric ulceration" OR "gastric ulcerations" OR<br>"esophagus ulcer" OR "esophagus ulcers" OR "esophagus ulceration" OR "esophagus ulcerations")
	("Curcumin" OR "Curcuma" OR "Curcuminoids" OR "turmeric" OR "diferuloylmethane")<br>("peptic ulcer disease" OR "duodenal ulcer" OR "duodenal ulcers" OR "duodenal ulceration" OR "duodenal ulcerations" OR

	"duodenum ulcer" OR "duodenum ulcers" OR "duodenum ulceration" OR "duodenum ulcerations" OR "gastrointestinal ulcer" OR "gastro-intestinal ulcer" OR "gastrointestinal ulcers" OR "gastro-intestinal ulcers" OR "gastrointestinal ulceration" OR "gastro-intestinal ulceration")
	("Curcumin" OR "Curcuma" OR "Curcuminoids" OR "turmeric" OR "diferuloylmethane") ("gastric mucosa" OR "gastritis" OR "chronic gastritis" OR "helicobacter pylori" OR "campylobacter pylori" OR "h. pylori")
	("Curcumin" OR "Curcuma" OR "Curcuminoids" OR "turmeric" OR "diferuloylmethane") ("gerd" OR "gastroesophageal reflux" OR "gastroesophageal reflux disease" OR "reflux esophagitis" OR "esophagitis" OR "dyspepsia" OR "functional dyspepsia" OR "epigastric pain" OR "gastroparesis" OR "hiatal hernia" OR "esophageal stricture" OR "barrett esophagus" OR "heartburn")
	(" Curcumin" OR "Curcuma" OR "Curcuminoids" OR "turmeric" OR "diferuloylmethane") ("gastrointestinal bleeding" OR "gastrointestinal hemorrhage" OR "gastrointestinal haemorrhage" OR "upper gastrointestinal bleeding" OR "UGIB" OR "peptic ulcer bleeding" OR "gastrointestinal perforation" OR "digestive perforation" OR "marginal ulcer" OR "curling ulcer")

Supplementary Table 2

Outcome	Subgroup	Number of studies	Groups		Heterogeneity		Effect estimates		
			Cur	Control	Chi^2	I^2 (%)	SMD	95% CI	*p*-value
Antioxidants									
CAT level	Overall	4	62	34	0.12	48.9	1.50	[0.80; 2.20]	< 0.0001
	Dosage								
	Dose of Cur > 18.5 mg/kg	4	62	34	0.12	48.9	1.50	[0.80; 2.20]	< 0.0001
	Dose of Cur ≤ 18.5 mg/kg	0	0	0	ND	ND	ND	ND	ND
	Approach								
	Protective approach	4	62	34	0.12	48.9	1.50	[0.80; 2.20]	< 0.0001
	Treatment approach	0	0	0	ND	ND	ND	ND	ND
	Administration route								
	Oral route	4	62	34	0.12	48.9	1.50	[0.80; 2.20]	< 0.0001
	Intraperitoneal route	0	0	0	ND	ND	ND	ND	ND
	Intraduodenal route	0	0	0	ND	ND	ND	ND	ND
	Induction method								
	NSAIDs-induced GU	1	24	8	ND	ND	ND	ND	ND
	Ethanol-induced GU	1	18	6	ND	ND	ND	ND	ND
	Stress-induced GU	1	10	10	ND	ND	ND	ND	ND
	Pyloric ligation-induced GU	0	0	0	ND	ND	ND	ND	ND
	Others induction methods	1	10	10	ND	ND	ND	ND	ND

Outcome	Subgroup	Number of studies	Groups		Heterogeneity		Effect estimates		
			Cur	Control	Chi^2	I^2 (%)	SMD	95% CI	*p*-value
GSH level	Overall	5	48	36	0.02	65.7	2.17	[1.05; 3.28]	0.0001
	Dosage								
	Dose of Cur > 18.5 mg/kg	5	48	36	0.02	65.7	2.17	[1.05; 3.28]	0.0001
	Dose of Cur ≤ 18.5 mg/kg	0	0	0	ND	ND	ND	ND	ND
	Approach								
	Protective approach	5	48	36	0.02	65.7	2.17	[1.05; 3.28]	0.0001
	Treatment approach	0	0	0	ND	ND	ND	ND	ND
	Administration route								
	Oral route	3	34	22	0.0042	81.8	2.94	[0.37; 5.51]	0.0252
	Intraperitoneal route	2	14	14	ND	ND	ND	ND	ND
	Intraduodenal route	0	0	0	ND	ND	ND	ND	ND
	Induction method								
	NSAIDs-induced GU	4	30	30	0.03	67.5	2.22	[0.54; 3.90]	0.0095
	Ethanol-induced GU	1	18	6	ND	ND	ND	ND	ND
	Stress-induced GU	0	0	0	ND	ND	ND	ND	ND
	Pyloric ligation-induced GU	0	0	0	ND	ND	ND	ND	ND
	Others induction methods	0	0	0	ND	ND	ND	ND	ND

Outcome	Subgroup	Number of studies	Groups		Heterogeneity		Effect estimates		
			Cur	Control	Chi^2	I^2 (%)	SMD	95% CI	*p*-value
SOD level	Overall	8	106	70	0.23	25.1	1.57	[1.15; 1.99]	< 0.0001
	Dosage								
	Dose of Cur > 18.5 mg/kg	8	98	68	0.32	13.7	1.65	[1.24; 2.06]	< 0.0001
	Dose of Cur ≤ 18.5 mg/kg	1	8	2	ND	ND	ND	ND	ND
	Approach								
	Protective approach	7	90	62	0.26	21.8	1.68	[1.22; 2.14]	< 0.0001
	Treatment approach	1	16	8	ND	ND	ND	ND	ND
	Administration route								
	Oral route	8	106	70	0.23	25.1	1.57	[1.15; 1.99]	< 0.0001
	Intraperitoneal route	0	0	0	ND	ND	ND	ND	ND
	Intraduodenal route	0	0	0	ND	ND	ND	ND	ND
	Induction method								
	NSAIDs-induced GU	2	34	18	ND	ND	ND	ND	ND
	Ethanol-induced GU	4	52	32	0.18	38.1	1.77	[1.06; 2.47]	< 0.0001
	Stress-induced GU	1	10	10	ND	ND	ND	ND	ND
	Pyloric ligation-induced GU	0	0	0	ND	ND	ND	ND	ND
	Others induction methods	1	10	10	ND	ND	ND	ND	ND

Outcome	Subgroup	Number of studies	Groups		Heterogeneity		Effect estimates		
			Cur	Control	Chi^2	I^2 (%)	SMD	95% CI	*p*-value
Oxidative stress									
MDA level	Overall	13	155	101	0.0002	68.2	-1.98	[-2.66; -1.30]	< 0.0001
	Dosage								
	Dose of Cur > 18.5 mg/kg	13	147	99	0.0002	68.1	-2.03	[-2.71; -1.34]	< 0.0001
	Dose of Cur ≤ 18.5 mg/kg	1	8	2	ND	ND	ND	ND	ND
	Approach								
	Protective approach	10	115	81	< 0.0001	73.5	-2.26	[-3.20; -1.33]	< 0.0001
	Treatment approach	3	40	20	0.28	20.9	-1.36	[-2.06; -0.65]	0.0002
	Administration route								
	Oral route	11	141	87	0.0001	71.5	-2.08	[-2.87; -1.28]	< 0.0001
	Intraperitoneal route	2	14	14	ND	ND	ND	ND	ND
	Intraduodenal route	0	0	0	ND	ND	ND	ND	ND
	Induction method								
	NSAIDs-induced GU	7	83	49	0.03	57.3	-1.73	[-2.42; -1.03]	< 0.0001
	Ethanol-induced GU	4	52	32	0.03	65.9	-1.87	[-2.88; -0.79]	0.0006
	Stress-induced GU	1	10	10	ND	ND	ND	ND	ND
	Pyloric ligation-induced GU	0	0	0	ND	ND	ND	ND	ND
	Others induction methods	1	10	10	ND	ND	ND	ND	ND

Outcome	Subgroup	Number of studies	Groups		Heterogeneity			Effect estimates	
			Cur	Control	Chi^2	I^2 (%)	SMD	95% CI	*p*-value
Inflammatory marker									
iNOS level	Overall	4	40	26	0.0011	81.4	-3.57	[-5.90; -1.24]	0.0027
	Dosage								
	Dose of Cur > 18.5 mg/kg	4	32	23	0.01	73.5	-4.56	[-7.26; -1.86]	0.0009
	Dose of Cur ≤ 18.5 mg/kg	2	8	3	ND	ND	ND	ND	ND
	Approach								
	Protective approach	2	22	14	ND	ND	ND	ND	ND
	Treatment approach	2	18	12	ND	ND	ND	ND	ND
	Administration route								
	Oral route	4	40	26	0.0011	81.4	-3.57	[-5.90; -1.24]	0.0027
	Intraperitoneal route	0	0	0	ND	ND	ND	ND	ND
	Intraduodenal route	0	0	0	ND	ND	ND	ND	ND
	Induction method								
	NSAIDs-induced GU	2	18	12	ND	ND	ND	ND	ND
	Ethanol-induced GU	0	0	0	ND	ND	ND	ND	ND
	Stress-induced GU	2	22	14	ND	ND	ND	ND	ND
	Pyloric ligation-induced GU	0	0	0	ND	ND	ND	ND	ND
	Others induction methods	0	0	0	ND	ND	ND	ND	ND

Outcome	Subgroup	Number of studies	Groups		Heterogeneity			Effect estimates	
			**Cur**	**Control**	**Chi^2**	**I^2 (%)**	**SMD**	**95% CI**	***p*-value**
TNF-α level	Overall	3	40	32	0.0008	85.9	-3.83	[-6.07; -1.60]	0.0008
	Dosage								
	Dose of Cur > 18.5 mg/kg	3	40	32	0.0008	85.9	-3.83	[-6.07; -1.60]	0.0008
	Dose of Cur ≤ 18.5 mg/kg	0	0	0	ND	ND	ND	ND	ND
	Approach								
	Protective approach	1	6	6	ND	ND	ND	ND	ND
	Treatment approach	2	34	26	ND	ND	ND	ND	ND
	Administration route								
	Oral route	3	40	32	0.0008	85.9	-3.83	[-6.07; -1.60]	0.0008
	Intraperitoneal route	0	0	0	ND	ND	ND	ND	ND
	Intraduodenal route	0	0	0	ND	ND	ND	ND	ND
	Induction method								
	NSAIDs-induced GU	2	24	24	ND	ND	ND	ND	ND
	Ethanol-induced GU	0	0	0	ND	ND	ND	ND	ND
	Stress-induced GU	0	0	0	ND	ND	ND	ND	ND
	Pyloric ligation-induced GU	0	0	0	ND	ND	ND	ND	ND
	Others induction methods	1	16	8	ND	ND	ND	ND	ND

NOTE: CI = confidence interval, CAT = catalase, Cur = curcumin, GSH = glutathione, GU = gastric ulcer, iNOS = inducible nitric oxide synthase, kg = kilogram, MDA = malondialdehyde, mg = milligram, ND = not determined, NSAIDs = nonsteroidal anti-inflammatory drugs, SMD = standard mean difference, SOD = superoxide dismutase, and TNF-α = tumor necrosis factor.

Supplementary Table 3

Outcome	Subgroup	Number of studies	Groups		Heterogeneity			Effect estimates	
			Cur	Control	Chi^2	I^2 (%)	SMD	95% CI	*p*-value
Acid output	Overall	8	189	71	0.01	64.8	-1.47	[-2.03; -0.92]	< 0.0001
	Dosage								
	Dose of Cur > 18.5 mg/kg	8	145	60	0.0019	69.1	-1.94	[-2.64; -1.24]	< 0.0001
	Dose of Cur ≤ 18.5 mg/kg	4	44	11	0.42	0.00	-0.82	[-1.52; -0.12]	0.0217
	Approach								
	Protective approach	4	90	40	0.0002	84.8	-1.71	[-2.89; 0.54]	0.0042
	Treatment approach	4	99	31	0.99	0.0	-1.34	[-1.80; -0.88]	< 0.0001
	Administration route								
	Oral route	4	59	31	0.15	44.3	-1.66	[-2.39; -0.93]	< 0.0001
	Intraperitoneal route	2	40	20	ND	ND	ND	ND	ND
	Intraduodenal route	2	90	20	ND	ND	ND	ND	ND
	Induction method								
	NSAIDs-induced GU	0	0	0	ND	ND	ND	ND	ND
	Ethanol-induced GU	0	0	0	ND	ND	ND	ND	ND
	Stress-induced GU	1	35	7	ND	ND	ND	ND	ND
	Pyloric ligation-induced GU	4	130	40	0.0043	77.2	-1.33	[-2.16; -0.49]	0.0019
	Others induction methods	3	24	24	0.08	60.8	-1.80	[-2.96; -0.65]	0.0023

Outcome	Subgroup	Number of studies	Groups		Heterogeneity			Effect estimates	
			Cur	Control	Chi^2	I^2 (%)	SMD	95% CI	*p*-value
Gastric acid volume	Overall	7	135	51	0.0006	74.8	-1.58	[-2.56; -0.61]	0.0015
	Dosage								
	Dose of Cur > 18.5 mg/kg	7	95	42	0.0041	68.5	-1.69	[-2.61; -0.77]	0.0003
	Dose of Cur ≤ 18.5 mg/kg	2	40	9	ND	ND	ND	ND	ND
	Approach								
	Protective approach	4	40	28	0.04	64.8	-2.53	[-3.71; -1.35]	< 0.0001
	Treatment approach	3	95	23	0.71	0.0	-2.63	[-1.09; -0.16]	0.0086
	Administration route								
	Oral route	5	45	31	0.0033	74.6	-2.06	[-3.37; -0.75]	0.0020
	Intraperitoneal route	0	0	0	ND	ND	ND	ND	ND
	Intraduodenal route	2	90	20	ND	ND	ND	ND	ND
	Induction method								
	NSAIDs-induced GU	1	6	6	ND	ND	ND	ND	ND
	Ethanol-induced GU	3	29	15	0.0010	85.5	-2.34	[-4.67; -0.01]	0.0494
	Stress-induced GU	0	0	0	ND	ND	ND	ND	ND
	Pyloric ligation-induced GU	3	100	30	0.57	0.0	-0.81	[-1.26; -0.37]	0.0003
	Others induction methods	0	0	0	ND	ND	ND	ND	ND

Outcome	Subgroup	Number of studies	Groups		Heterogeneity		Effect estimates		
			Cur	Control	Chi^2	I^2 (%)	SMD	95% CI	*p*-value
Gastrin level	Overall	3	79	22	< 0.0001	93.4	0.62	[-1.52; 2.76]	0.5690
	Dosage								
	Dose of Cur > 18.5 mg/kg	3	51	15	< 0.0001	95.1	1.60	[-1.63; 4.82]	0.3325
	Dose of Cur ≤ 18.5 mg/kg	2	28	7	ND	ND	ND	ND	ND
	Approach								
	Protective approach	2	63	14	ND	ND	ND	ND	ND
	Treatment approach	1	16	8	ND	ND	ND	ND	ND
	Administration route								
	Oral route	3	79	22	< 0.0001	93.4	0.62	[-1.52; 2.76]	0.5690
	Intraperitoneal route	0	0	0	ND	ND	ND	ND	ND
	Intraduodenal route	0	0	0	ND	ND	ND	ND	ND
	Induction method								
	NSAIDs-induced GU	0	0	0	ND	ND	ND	ND	ND
	Ethanol-induced GU	1	28	7	ND	ND	ND	ND	ND
	Stress-induced GU	1	35	7	ND	ND	ND	ND	ND
	Pyloric ligation-induced GU	0	0	0	ND	ND	ND	ND	ND
	Others induction methods	1	16	8	ND	ND	ND	ND	ND

Outcome	Subgroup	Number of studies	Groups		Heterogeneity			Effect estimates	
			Cur	Control	Chi^2	I^2 (%)	SMD	95% CI	*p*-value
H^+/K^+–ATPase level	Overall	3	23	23	0.17	43.7	-1.97	[-2.98; -0.95]	0.0001
	Dosage								
	Dose of Cur > 18.5 mg/kg	3	23	23	0.17	43.7	-1.97	[-2.98; -0.95]	0.0001
	Dose of Cur ≤ 18.5 mg/kg	0	0	0	ND	ND	ND	ND	ND
	Approach								
	Protective approach	3	23	23	0.17	43.7	-1.97	[-2.98; -0.95]	0.0001
	Treatment approach	0	0	0	ND	ND	ND	ND	ND
	Administration route								
	Oral route	3	23	23	0.17	43.7	-1.97	[-2.98; -0.95]	0.0001
	Intraperitoneal route	0	0	0	ND	ND	ND	ND	ND
	Intraduodenal route	0	0	0	ND	ND	ND	ND	ND
	Induction method								
	NSAIDs-induced GU	0	0	0	ND	ND	ND	ND	ND
	Ethanol-induced GU	1	10	10	ND	ND	ND	ND	ND
	Stress-induced GU	2	13	13	ND	ND	ND	ND	ND
	Pyloric ligation-induced GU	0	0	0	ND	ND	ND	ND	ND
	Others induction methods	0	0	0	ND	ND	ND	ND	ND

Outcome	Subgroup	Number of studies	Groups		Heterogeneity			Effect estimates	
			Cur	Control	Chi^2	I^2 (%)	SMD	95% CI	*p*-value
pH of gastric juice	Overall	4	27	25	0.04	63.0	1.32	[0.07; 2.58]	0.0386
	Dosage								
	Dose of Cur > 18.5 mg/kg	4	27	25	0.04	63.0	1.32	[0.07; 2.58]	0.0386
	Dose of Cur ≤ 18.5 mg/kg	0	0	0	ND	ND	ND	ND	ND
	Approach								
	Protective approach	3	22	22	0.02	75.4	1.60	[-0.37; 3.58]	0.115
	Treatment approach	1	5	3	ND	ND	ND	ND	ND
	Administration route								
	Oral route	4	27	25	0.04	63.0	1.32	[0.07; 2.58]	0.0386
	Intraperitoneal route	0	0	0	ND	ND	ND	ND	ND
	Intraduodenal route	0	0	0	ND	ND	ND	ND	ND
	Induction method								
	NSAIDs-induced GU	1	6	6	ND	ND	ND	ND	ND
	Ethanol-induced GU	2	11	9	ND	ND	ND	ND	ND
	Stress-induced GU	1	10	10	ND	ND	ND	ND	ND
	Pyloric ligation-induced GU	0	0	0	ND	ND	ND	ND	ND
	Others induction methods	0	0	0	ND	ND	ND	ND	ND

Outcome	Subgroup	Number of studies	Groups		Heterogeneity			Effect estimates	
			Cur	Control	Chi^2	I^2 (%)	SMD	95% CI	*p*-value
Total acidity	Overall	7	160	58	< 0.0001	87.4	-1.77	[-2.83; -0.71]	0.0011
	Dosage								
	Dose of Cur > 18.5 mg/kg	7	120	49	< 0.0001	84.9	-1.78	[-2.80; -0.77]	0.0006
	Dose of Cur ≤ 18.5 mg/kg	2	40	9	ND	ND	ND	ND	ND
	Approach								
	Protective approach	5	80	38	< 0.0001	90.5	-20.11	[-58.82; 18.59]	0.3084
	Treatment approach	2	80	20	ND	ND	ND	ND	ND
	Administration route								
	Oral route	5	70	38	0.0001	82.6	-2.35	[-2.94; -1.77]	< 0.0001
	Intraperitoneal route	0	0	0	ND	ND	ND	ND	ND
	Intraduodenal route	2	90	20	ND	ND	ND	ND	ND
	Induction method								
	NSAIDs-induced GU	1	6	6	ND	ND	ND	ND	ND
	Ethanol-induced GU	2	24	12	ND	ND	ND	ND	ND
	Stress-induced GU	0	0	0	ND	ND	ND	ND	ND
	Pyloric ligation-induced GU	4	130	40	< 0.0001	87.8	-1.62	[-3.11; -0.13]	0.0329
	Others induction methods	0	0	0	ND	ND	ND	ND	ND

NOTE: CI = confidence interval, Cur = curcumin, GU = gastric ulcer, H^+K^+ ATPase = hydrogen-potassium adenosine triphosphatase, kg = kilogram, mg = milligram, ND = not determined, NSAIDs = nonsteroidal anti-inflammatory drugs, pH = potential of hydrogen, SMD = standard mean difference.

Supplementary Table 4

Outcome	Subgroup	Number of studies	Groups		Heterogeneity		Effect estimates		
			Cur	Control	Chi^2	I^2 (%)	SMD	95% CI	*p*-value
Ulcer area	Overall	4	99	28	0.95	0.0	-1.21	[-1.66; -0.75]	< 0.0001
	Dosage								
	Dose of Cur > 18.5 mg/kg	4	63	19	0.02	68.9	-2.49	[-3.78; -1.19]	0.0002
	Dose of Cur ≤ 18.5 mg/kg	3	36	9	0.76	0.0	-0.44	[-1.18; 0.30]	0.2479
	Approach								
	Protective approach	4	99	28	0.95	0.0	-1.21	[-1.66; -0.75]	< 0.0001
	Treatment approach	0	0	0	ND	ND	ND	ND	ND
	Administration route								
	Oral route	4	99	28	0.95	0.0	-1.21	[-1.66; -0.75]	< 0.0001
	Intraperitoneal route	0	0	0	ND	ND	ND	ND	ND
	Intraduodenal route	0	0	0	ND	ND	ND	ND	ND
	Induction method								
	NSAIDs-induced GU	2	59	15	ND	ND	ND	ND	ND
	Ethanol-induced GU	1	28	7	ND	ND	ND	ND	ND
	Stress-induced GU	1	12	6	ND	ND	ND	ND	ND
	Pyloric ligation-induced GU	0	0	0	ND	ND	ND	ND	ND
	Others induction methods	0	0	0	ND	ND	ND	ND	ND

Outcome	Subgroup	Number of studies	Groups		Heterogeneity		Effect estimates		
			Cur	Control	Chi^2	I^2 (%)	SMD	95% CI	*p*-value
Ulcer index	Overall	24	530	224	< 0.0001	73.1	-2.19	[-2.64; -1.73]	< 0.0001
	Dosage								
	Dose of Cur > 18.5mg/kg	24	421	193	< 0.0001	72.0	-2.40	[-2.90; -1.91]	< 0.0001
	Dose of Cur ≤ 18.5 mg/kg	7	109	31	0.04	55.1	-1.34	[-2.02; -0.66]	0.0001
	Approach								
	Protective approach	18	397	167	< 0.0001	70.7	-2.08	[-2.58; -1.58]	< 0.0001
	Treatment approach	6	133	57	< 0.0001	81.0	-3.01	[-4.93; -1.09]	0.0021
	Administration route								
	Oral route	19	374	172	< 0.0001	75.8	-2.42	[-3.04; -1.81]	< 0.0001
	Intraperitoneal route	5	156	52	0.08	51.9	-1.64	[-2.18; -1.11]	< 0.0001
	Intraduodenal route	0	0	0	ND	ND	ND	ND	ND
	Induction method								
	NSAIDs-induced GU	10	279	95	0.0003	71.1	-1.76	[-2.16; -1.36]	< 0.0001
	Ethanol-induced GU	5	107	45	0.13	43.8	-2.71	[-3.41; -2.02]	< 0.0001
	Stress-induced GU	4	72	36	0.0016	80.3	-2.04	[-3.81; -0.27]	0.0241
	Pyloric ligation-induced GU	2	40	20	ND	ND	ND	ND	ND
	Others induction methods	3	32	28	0.0017	84.3	-2.56	[-4.96; -0.16]	0.0367

Outcome	Subgroup	Number of studies	Groups		Heterogeneity		Effect estimates		
			Cur	Control	Chi^2	I^2 (%)	SMD	95% CI	*p*-value
Mucosa healing index	Overall	4	104	34	0.24	29.4	1.20	[0.78; 1.63]	< 0.0001
	Dosage								
	Dose of Cur > 18.5 mg/kg	4	84	29	0.26	24.7	1.27	[0.78; 1.77]	< 0.0001
	Dose of Cur ≤ 18.5 mg/kg	1	20	5	ND	ND	ND	ND	ND
	Approach								
	Protective approach	1	40	10	ND	ND	ND	ND	ND
	Treatment approach	3	64	24	0.14	49.2	1.36	[0.61; 2.12]	0.0004
	Administration route								
	Oral route	4	104	34	0.24	29.4	1.20	[0.78; 1.63]	< 0.0001
	Intraperitoneal route	0	0	0	ND	ND	ND	ND	ND
	Intraduodenal route	0	0	0	ND	ND	ND	ND	ND
	Induction method								
	NSAIDs-induced GU	4	104	34	0.24	29.4	1.20	[0.78; 1.63]	< 0.0001
	Ethanol-induced GU	0	0	0	ND	ND	ND	ND	ND
	Stress-induced GU	0	0	0	ND	ND	ND	ND	ND
	Pyloric ligation-induced GU	0	0	0	ND	ND	ND	ND	ND
	Others induction methods	0	0	0	ND	ND	ND	ND	ND

Outcome	Subgroup	Number of studies	Groups		Heterogeneity			Effect estimates	
			Cur	Control	Chi^2	I^2 (%)	SMD	95% CI	*p*-value
Ulcer healing index	Overall	4	104	34	0.0027	78.9	1.53	[0.13; 2.92]	0.0318
	Dosage								
	Dose of Cur > 18.5 mg/kg	4	84	29	0.01	74.4	1.61	[0.30; 2.92]	0.0163
	Dose of Cur ≤ 18.5 mg/kg	1	20	5	ND	ND	ND	ND	ND
	Approach								
	Protective approach	1	40	10	ND	ND	ND	ND	ND
	Treatment approach	3	64	24	0.0040	81.9	1.95	[0.09; 3.81]	0.0403
	Administration route								
	Oral route	3	74	24	0.0010	85.6	1.85	[-0.17; 3.88]	0.0728
	Intraperitoneal route	0	0	0	ND	ND	ND	ND	ND
	Intraduodenal route	1	30	10	ND	ND	ND	ND	ND
	Induction methods								
	NSAIDs-induced GU	2	42	16	ND	ND	ND	ND	ND
	Ethanol-induced GU	1	40	10	ND	ND	ND	ND	ND
	Stress-induced GU	1	22	8	ND	ND	ND	ND	ND
	Pyloric ligation-induced GU	0	0	0	ND	ND	ND	ND	ND
	Others induction methods	0	0	0	ND	ND	ND	ND	ND

NOTE: CI = confidence interval, Cur = curcumin, GU = gastric ulcer, kg = kilogram, mg = milligram, ND = not determined, NSAIDs = nonsteroidal anti-inflammatory drugs, and SMD = standard mean difference.

Supplementary Table 5

Outcome	Subgroup	Number of studies	Groups		Heterogeneity			Effect estimates	
			Cur	Control	Chi²	I² (%)	SMD	95% CI	*p*-value
Blood glucose level	Overall	4	42	26	0.0039	77.5	-2.67	[-5.00; -0.34]	0.0248
	Dosage								
	Dose of Cur > 18.5 mg/kg	4	42	26	0.0039	77.5	-2.67	[-5.00; -0.34]	0.0248
	Dose of Cur ≤ 18.5 mg/kg	0	0	0	ND	ND	ND	ND	ND
	Approach								
	Protective approach	0	0	0	ND	ND	ND	ND	ND
	Treatment approach	4	42	26	0.0039	77.5	-2.67	[-5.00; -0.34]	0.0248
	Administration route								
	Oral route	4	42	26	0.0039	77.5	-2.67	[-5.00; -0.34]	0.0248
	Intraperitoneal route	0	0	0	ND	ND	ND	ND	ND
	Intraduodenal route	0	0	0	ND	ND	ND	ND	ND
	Induction method								
	HFD-induced diabetic gastroparesis	2	8	8	ND	ND	ND	ND	ND
	STZ-induced diabetic gastroparesis	2	34	18	ND	ND	ND	ND	ND

Outcome	Subgroup	Number of studies	Groups		Heterogeneity			Effect estimates	
			**Cur**	**Control**	**Chi^2**	**I^2 (%)**	**SMD**	**95% CI**	***p*-value**
Gastric emptying rate	Overall	4	42	26	0.32	14.7	1.69	[1.04; 2.34]	< 0.0001
	Dosage								
	Dose of Cur > 18.5 mg/kg	4	42	26	0.32	14.7	1.69	[1.04; 2.34]	< 0.0001
	Dose of Cur ≤ 18.5 mg/kg	0	0	0	ND	ND	ND	ND	ND
	Approach								
	Protective approach	0	0	0	ND	ND	ND	ND	ND
	Treatment approach	4	42	26	0.32	14.7	1.69	[1.04; 2.34]	< 0.0001
	Administration route								
	Oral route	4	42	26	0.32	14.7	1.69	[1.04; 2.34]	< 0.0001
	Intraperitoneal route	0	0	0	ND	ND	ND	ND	ND
	Intraduodenal route	0	0	0	ND	ND	ND	ND	ND
	Induction method								
	HFD-induced diabetic gastroparesis	2	8	8	ND	ND	ND	ND	ND
	STZ-induced diabetic gastroparesis	2	34	18	ND	ND	ND	ND	ND

NOTE: CI = confidence interval, Cur = curcumin, HFD = high fat diet, kg = kilogram, mg = milligram, ND = not determined, SMD = standard mean difference, and STZ = streptozocin.

Supplementary Fig. 1

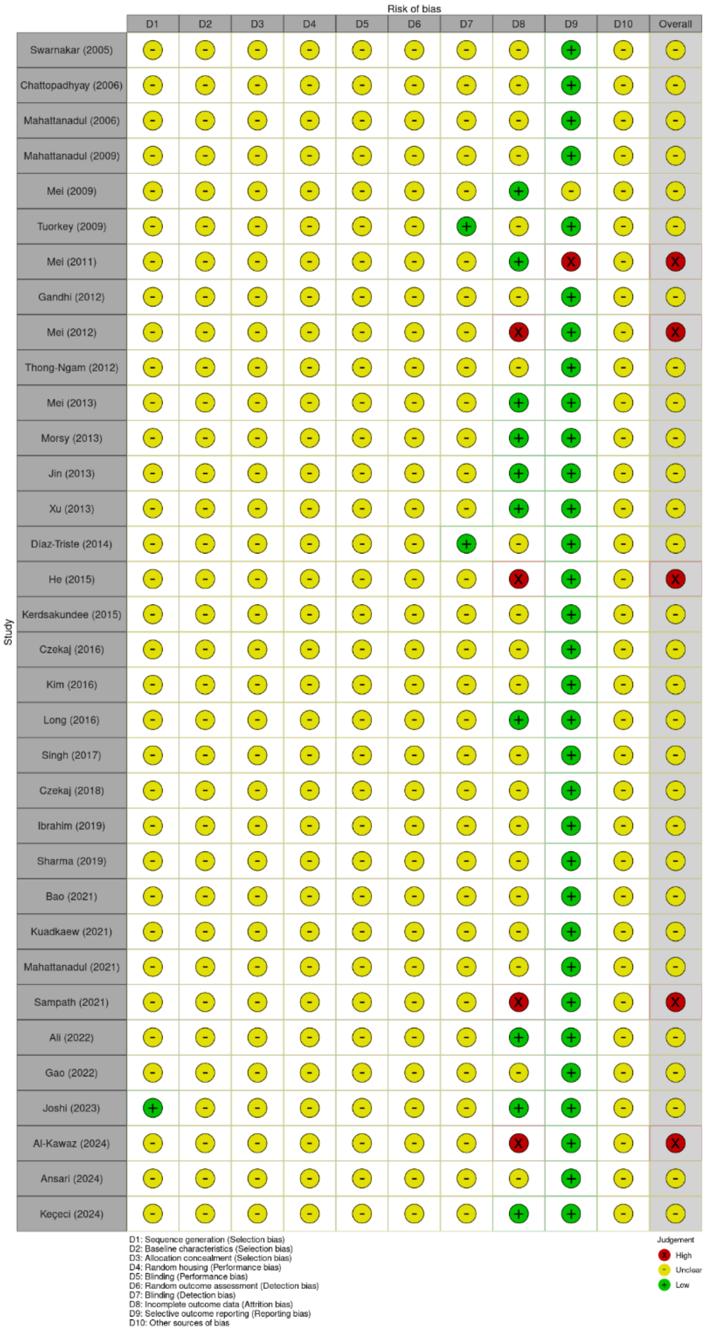

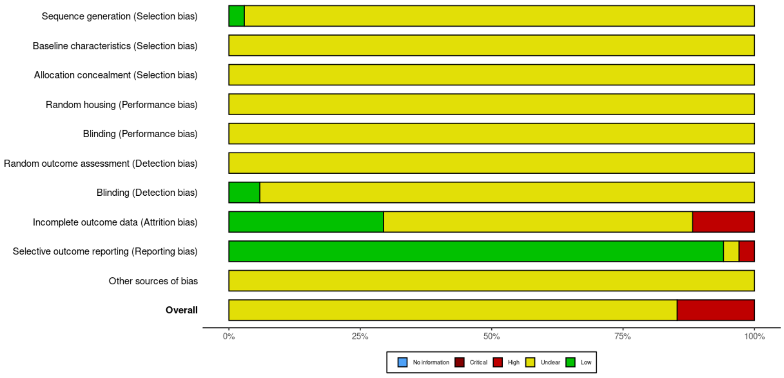

Supplementary Fig. 2

CAT level

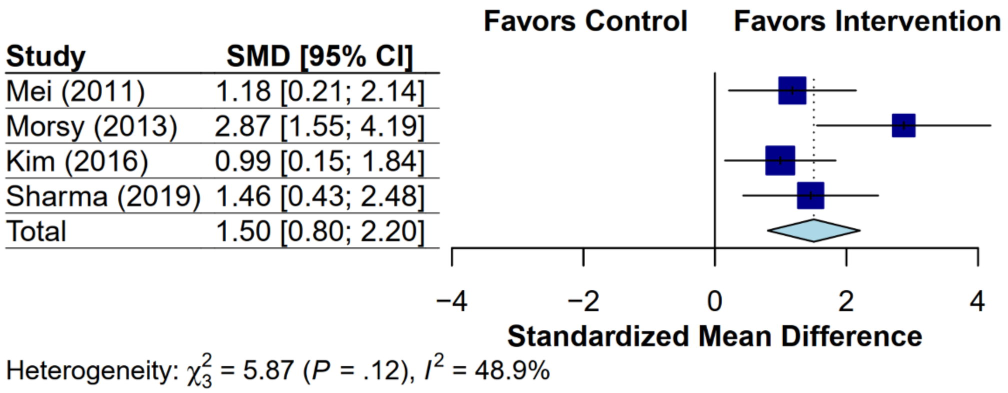

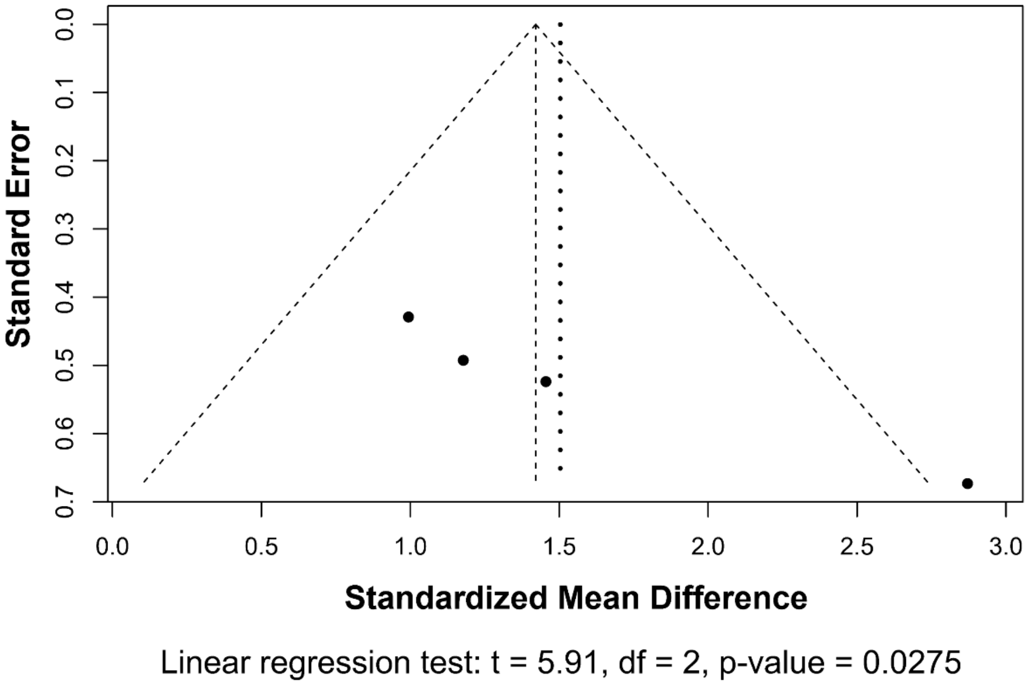

Supplementary Fig. 3

GSH level

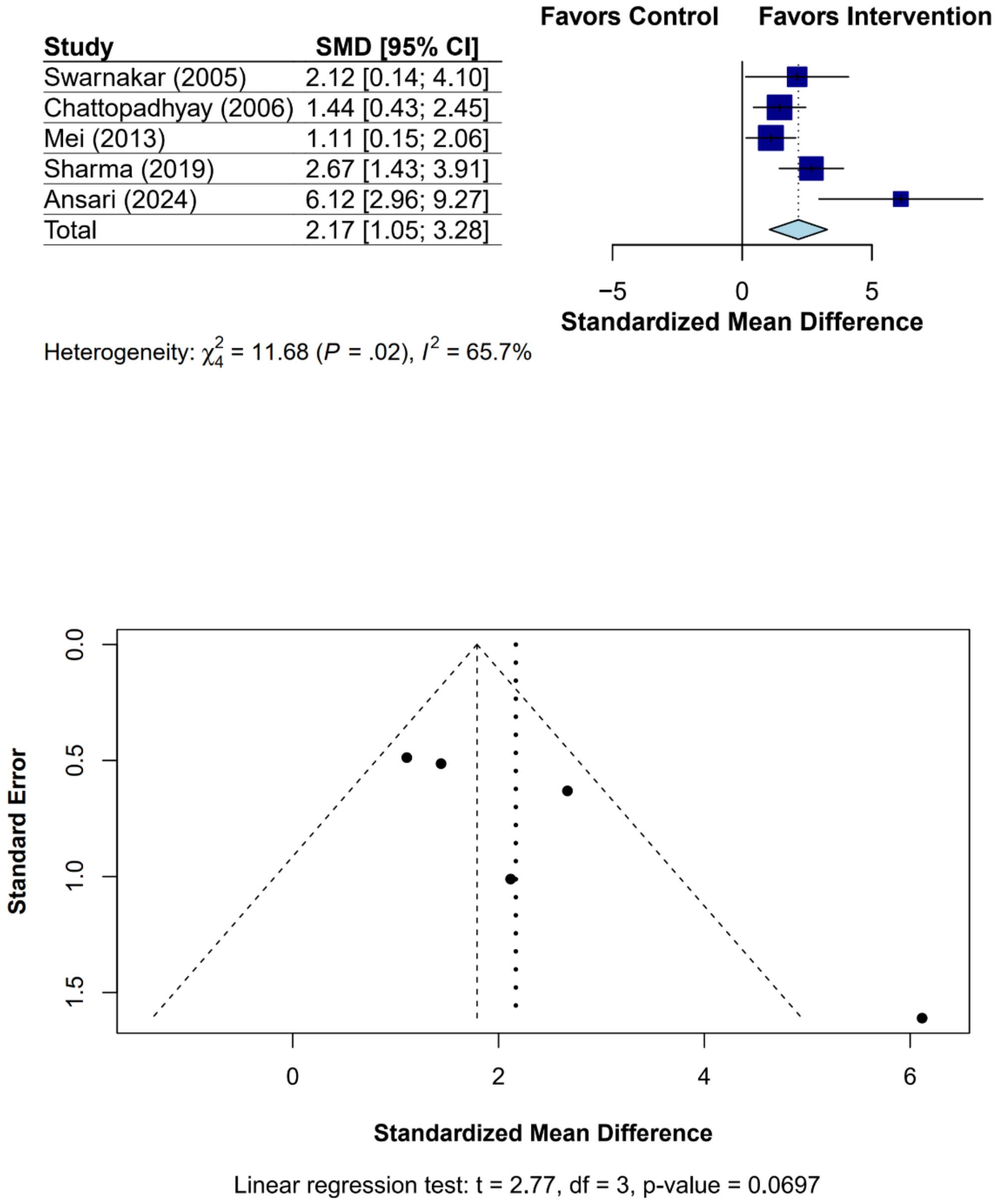

Supplementary Fig. 4

SOD level

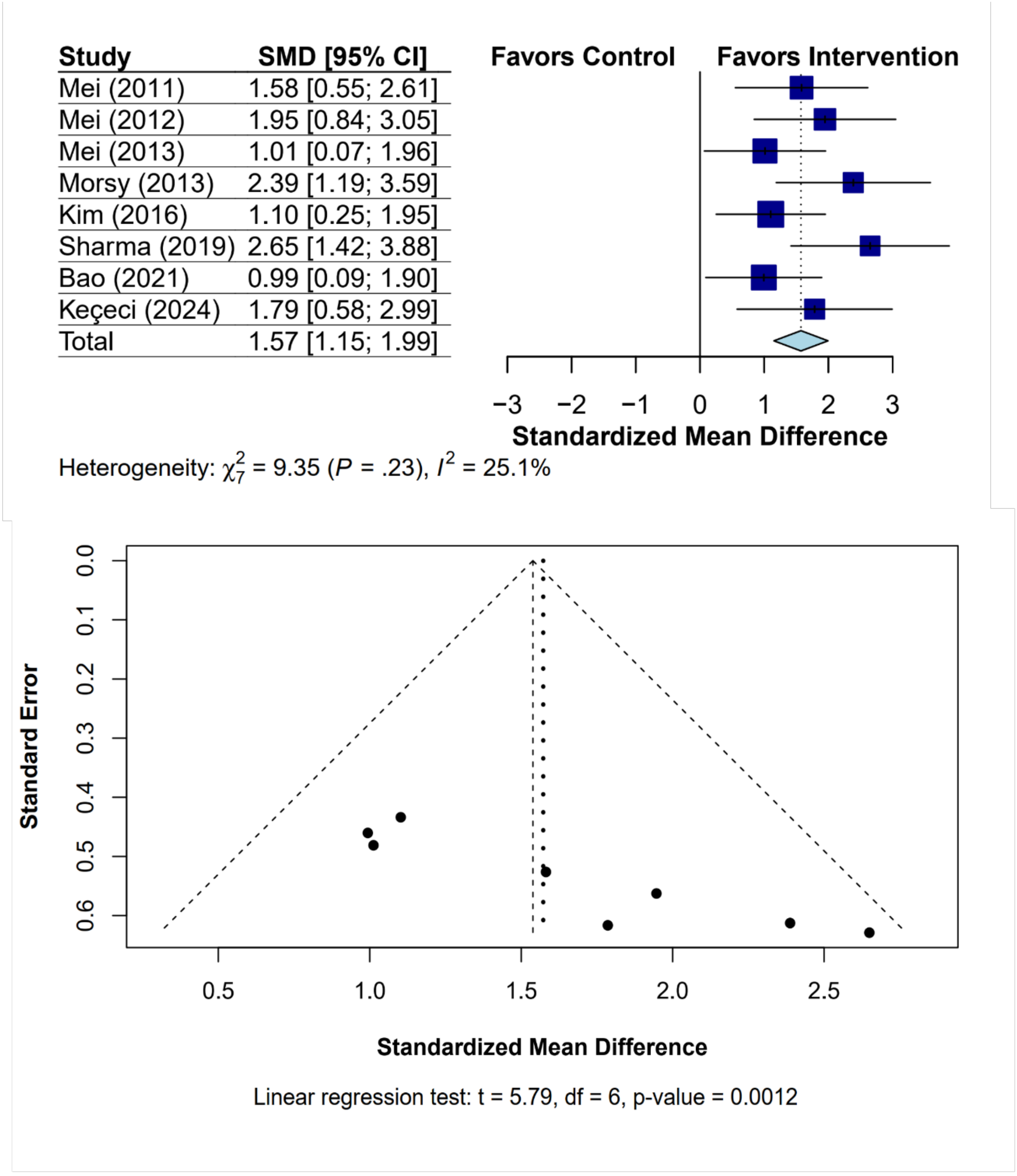

Supplementary Fig. 5

MDA level

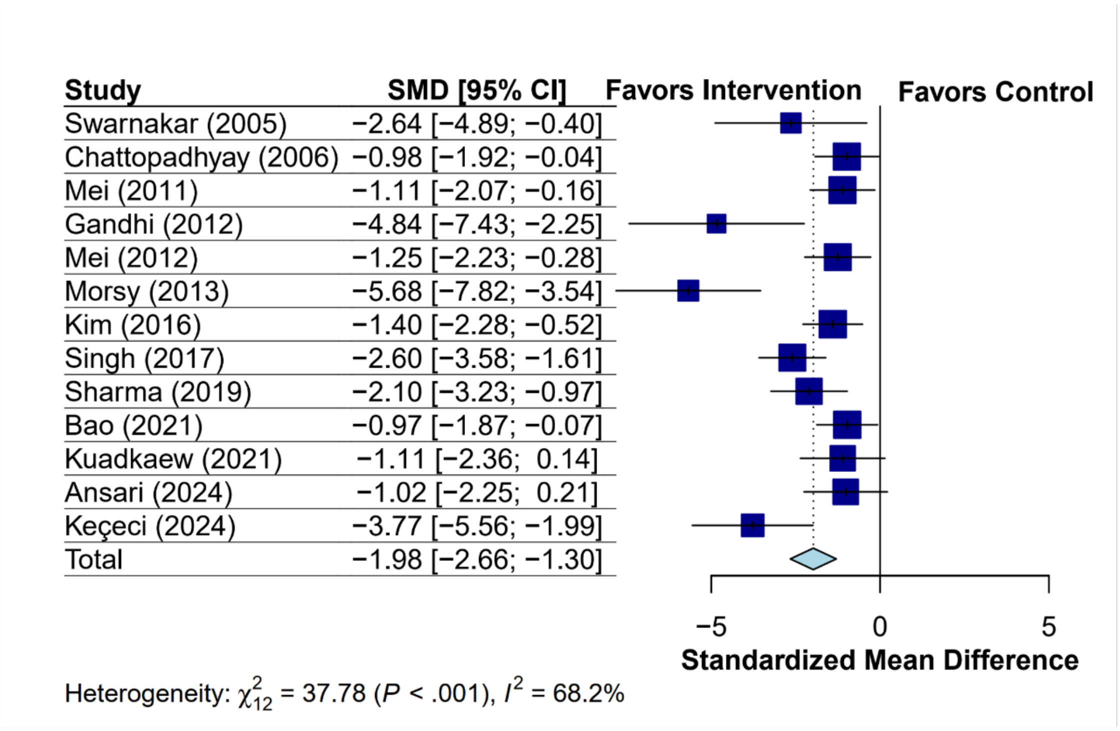

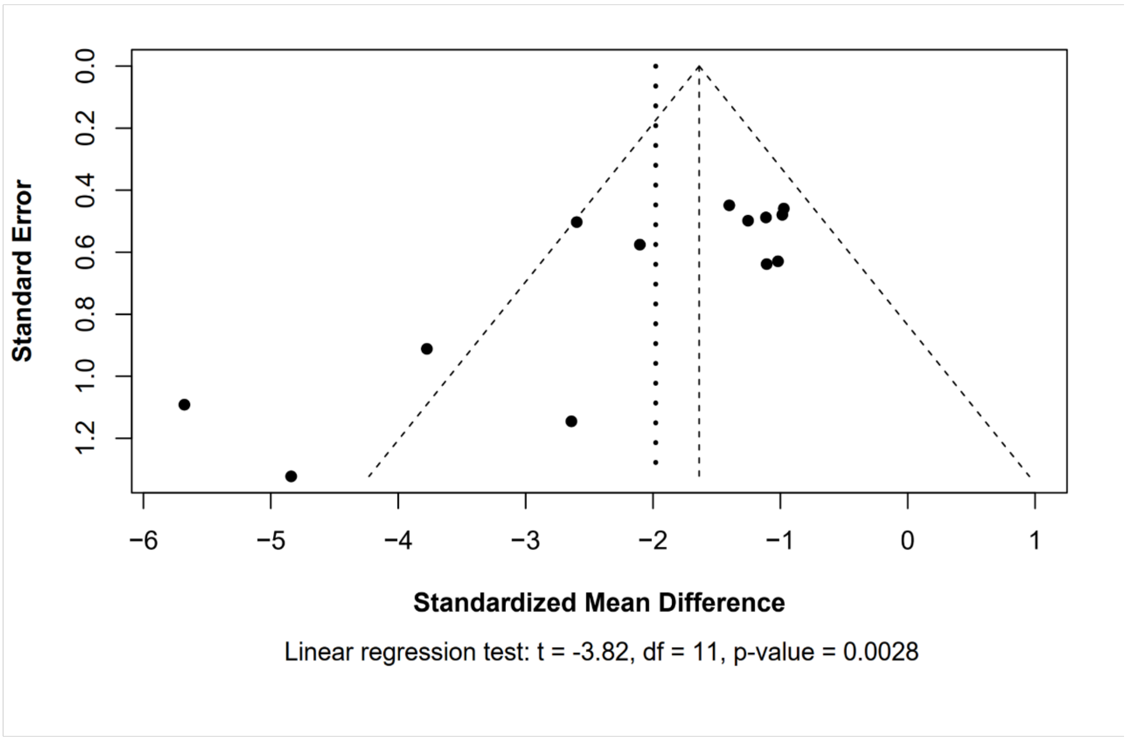

Supplementary Fig. 6

iNOS level

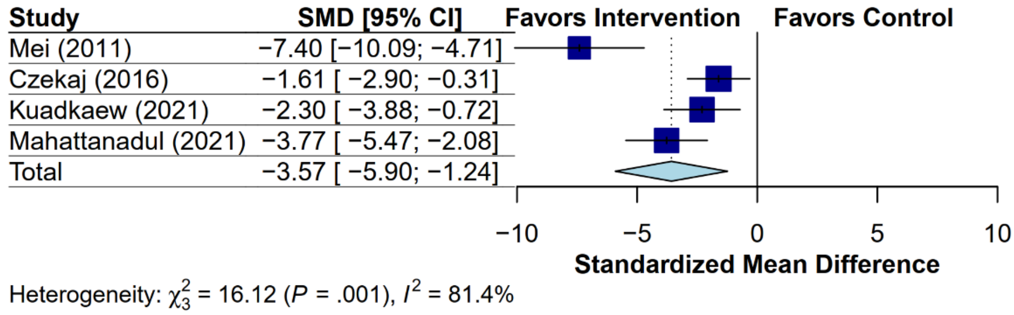

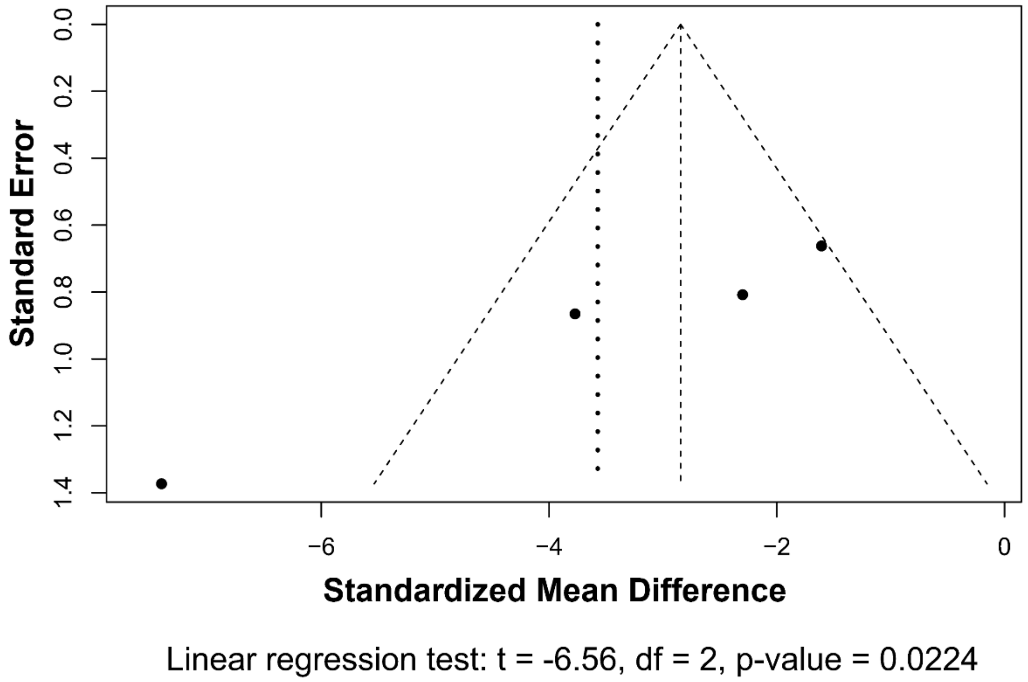

Supplementary Fig. 7

TNF-α level

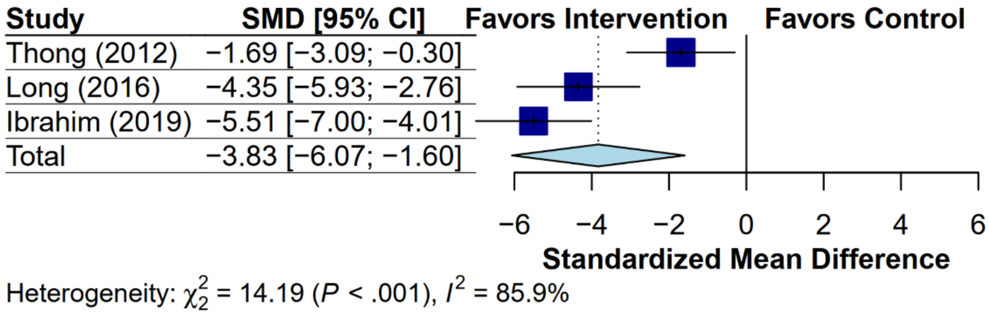

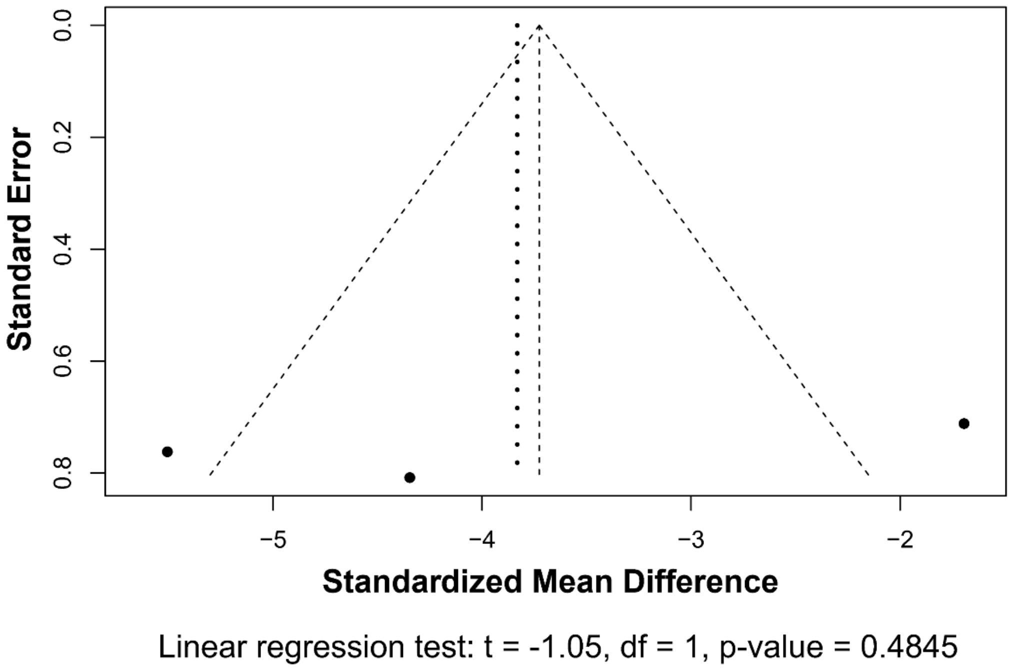

Supplementary Fig. 8

Acid output

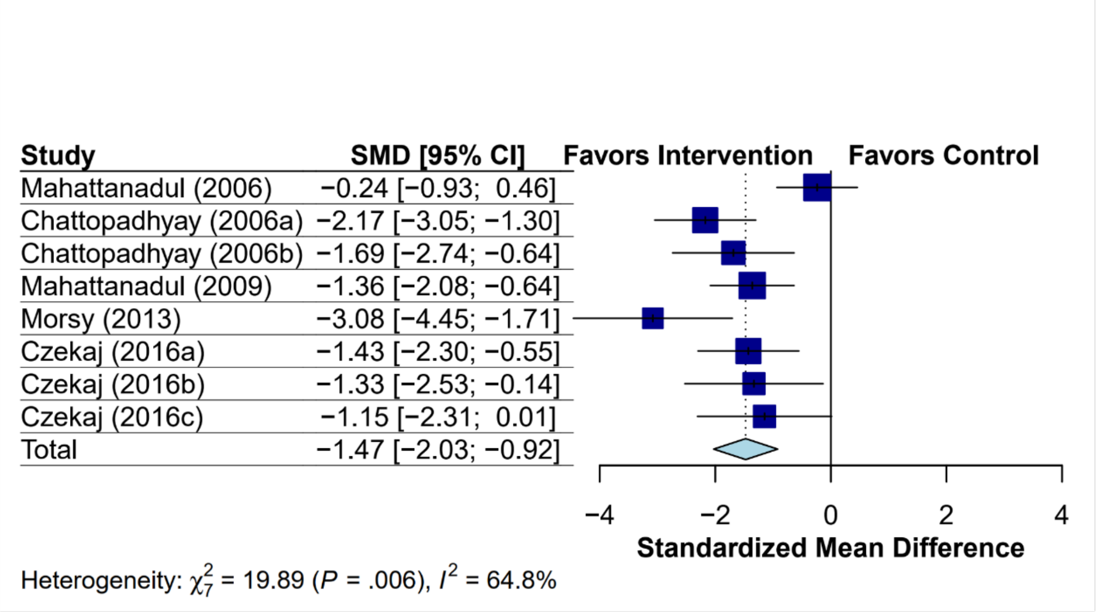

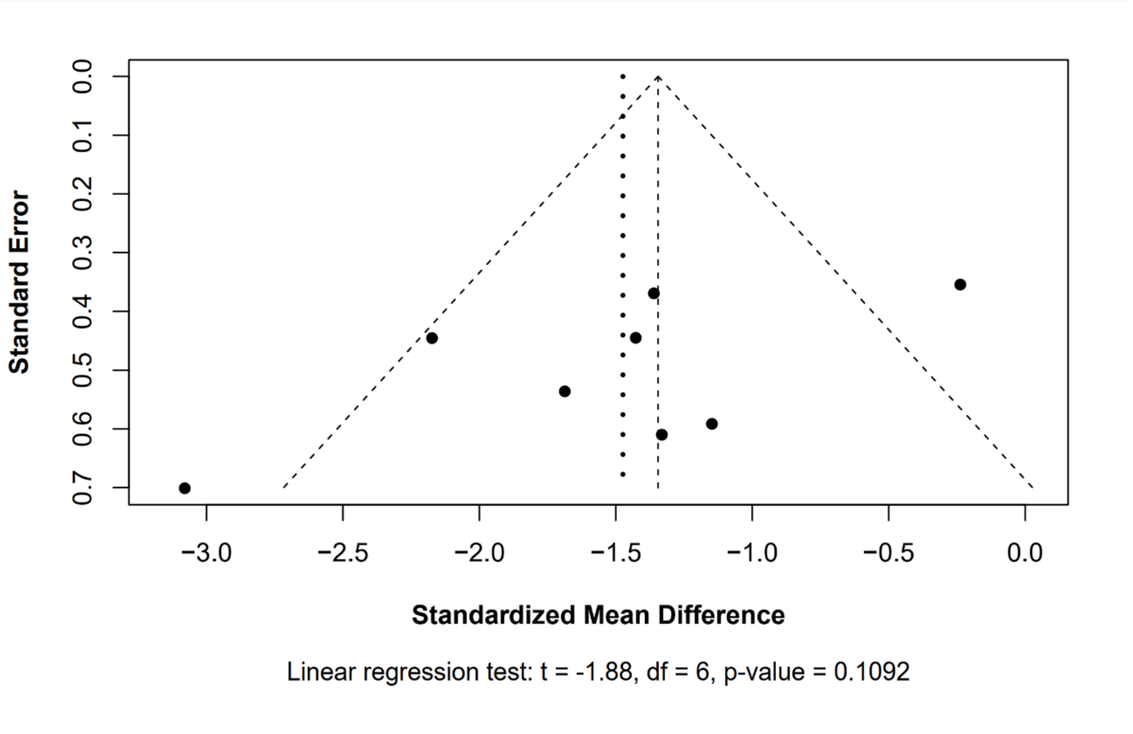

Supplementary Fig. 9

Gastric acid volume

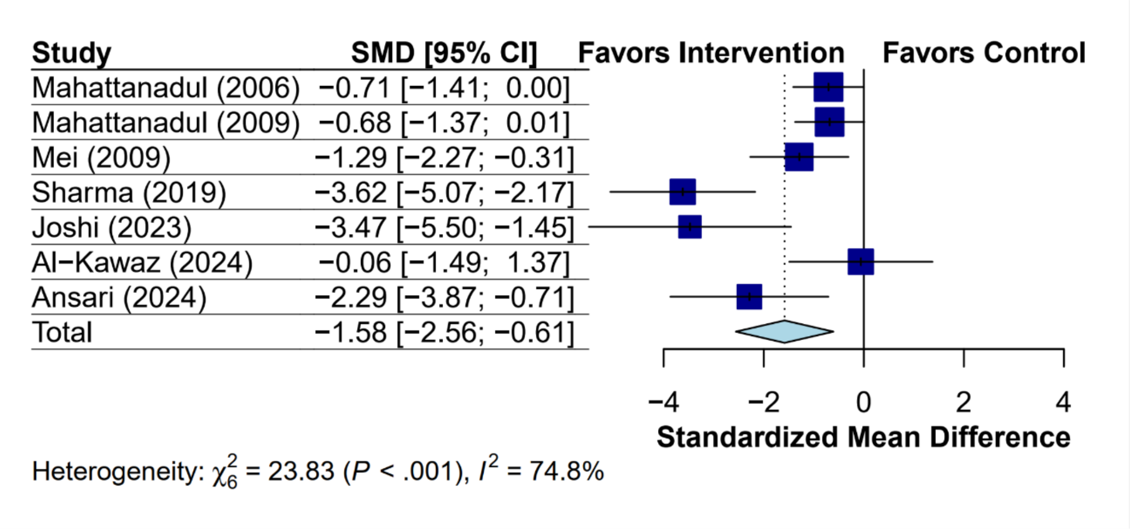

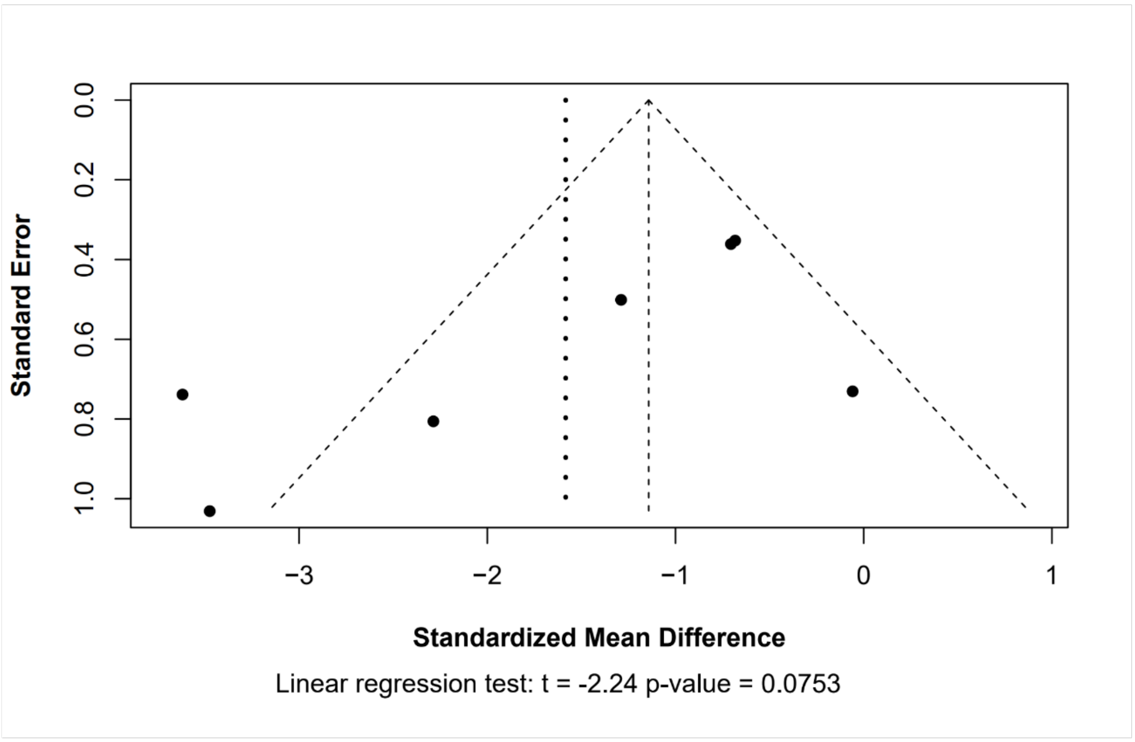

Supplementary Fig. 10

Gastrin level

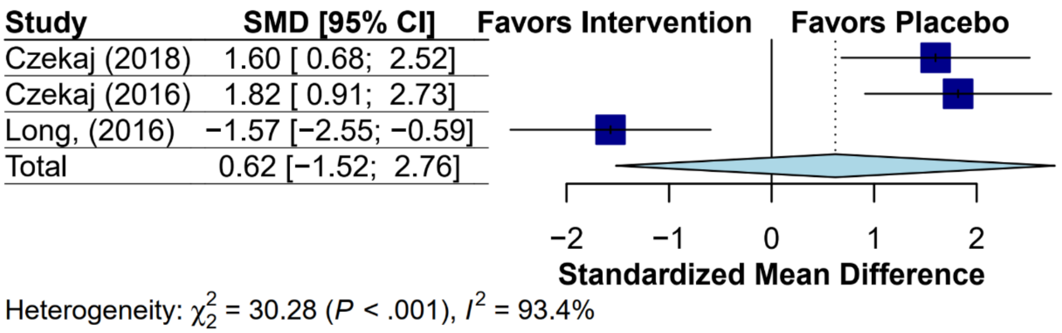

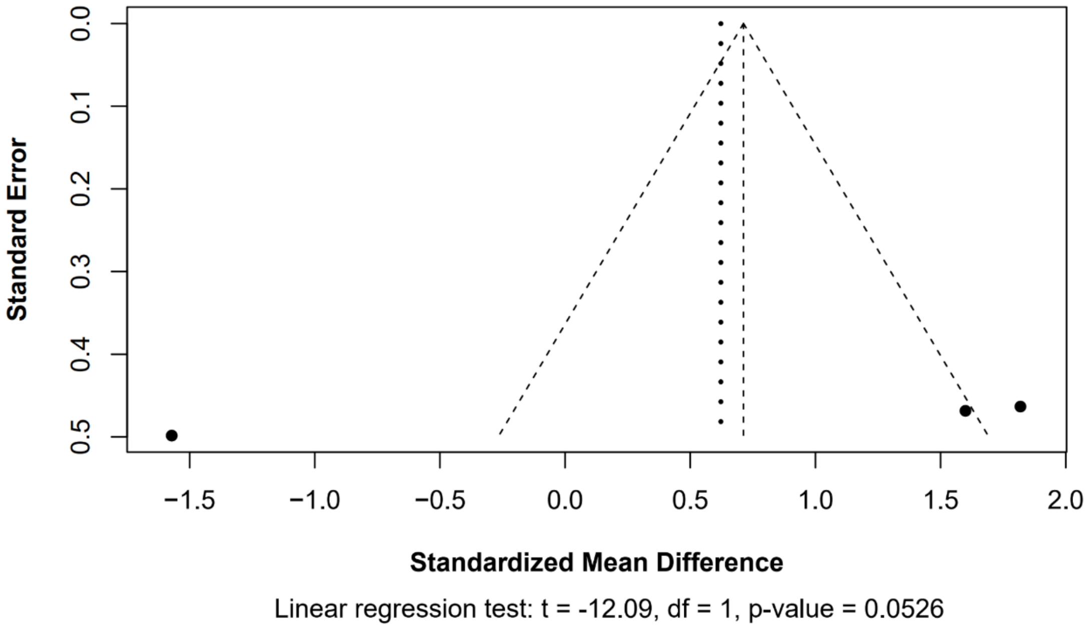

Supplementary Fig. 11

H $^{+}$/K^{+} -ATPase level

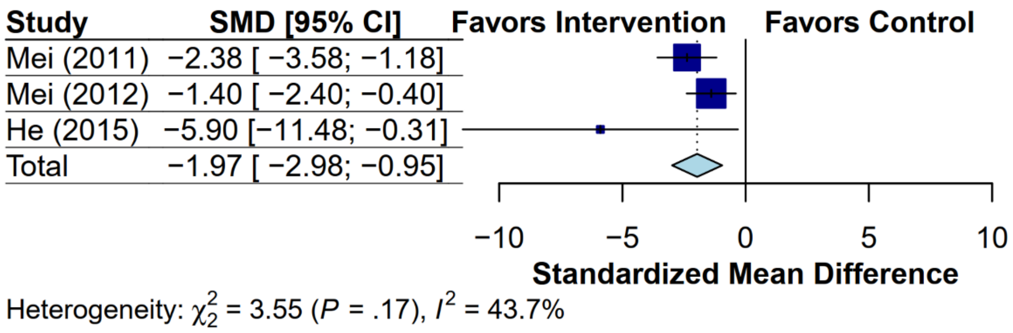

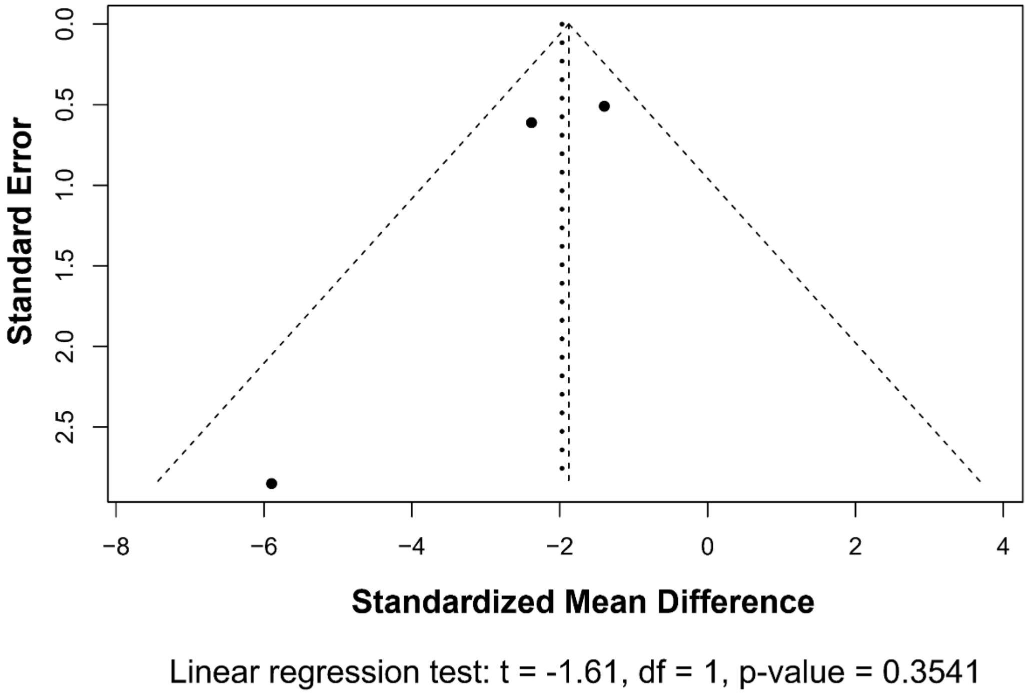

Supplementary Fig. 12

pH of gastric acid

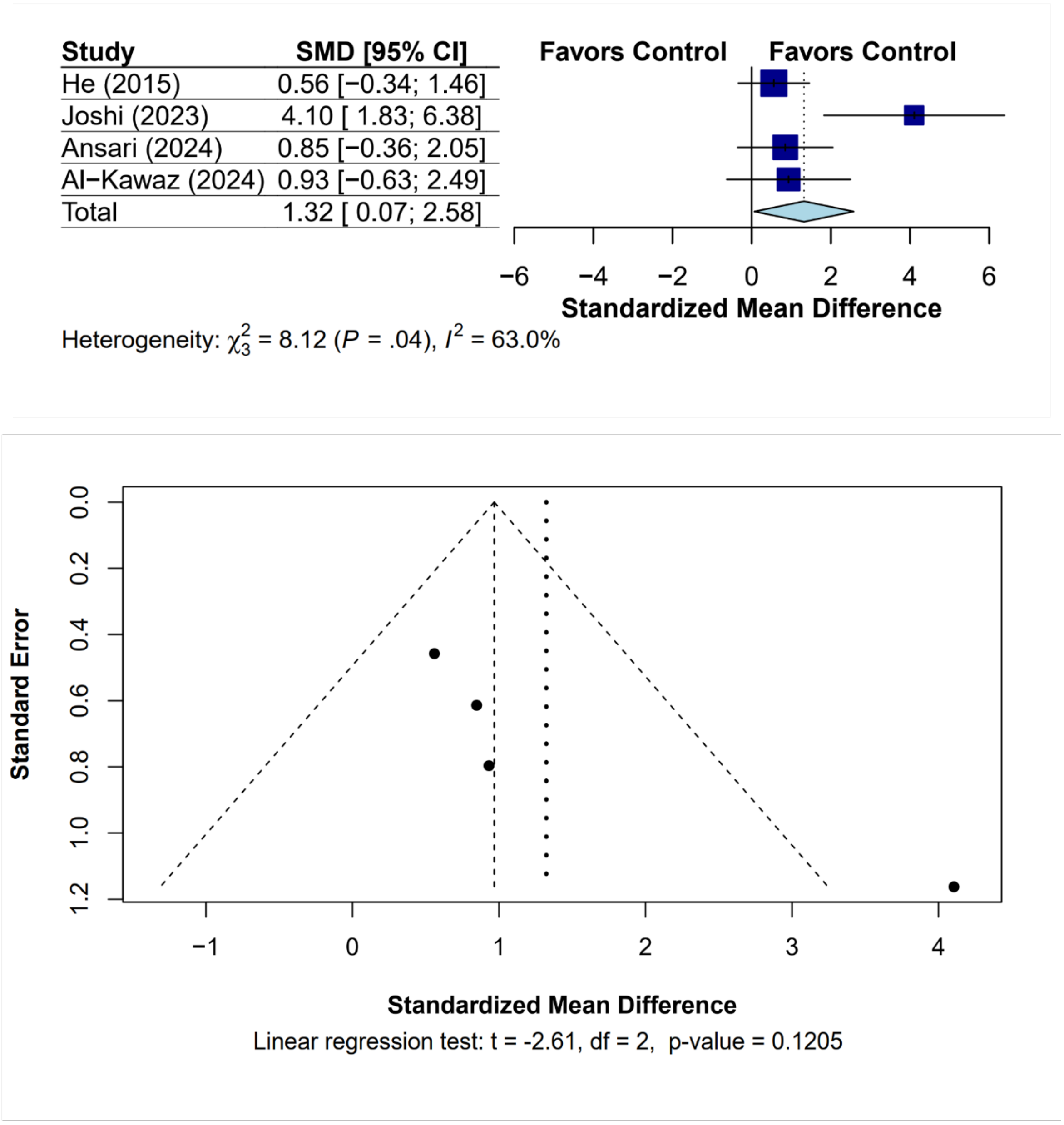

Supplementary Fig. 13

Total acidity

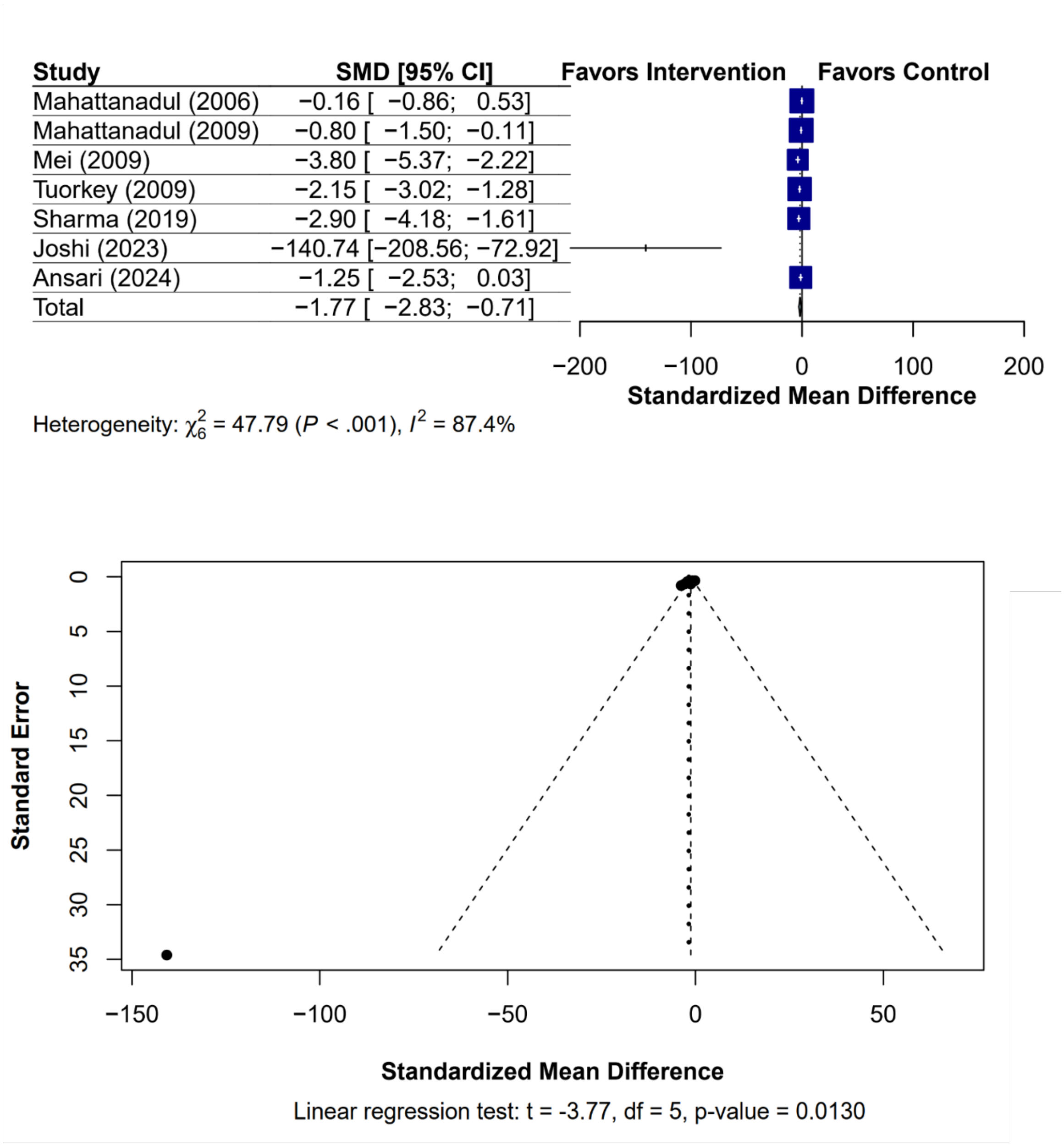

Supplementary Fig. 14

Ulcer area

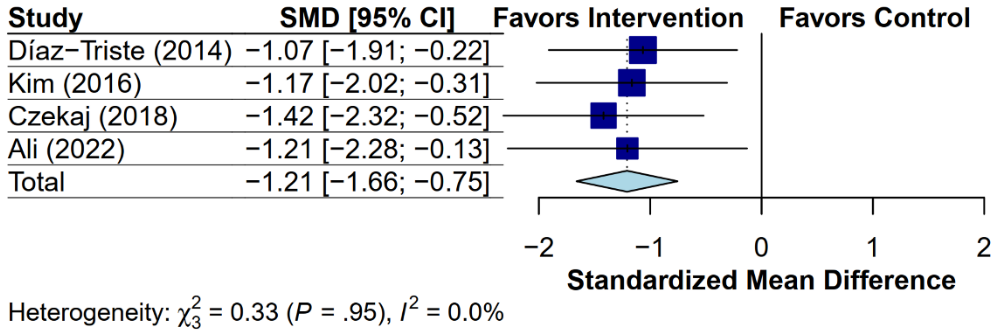

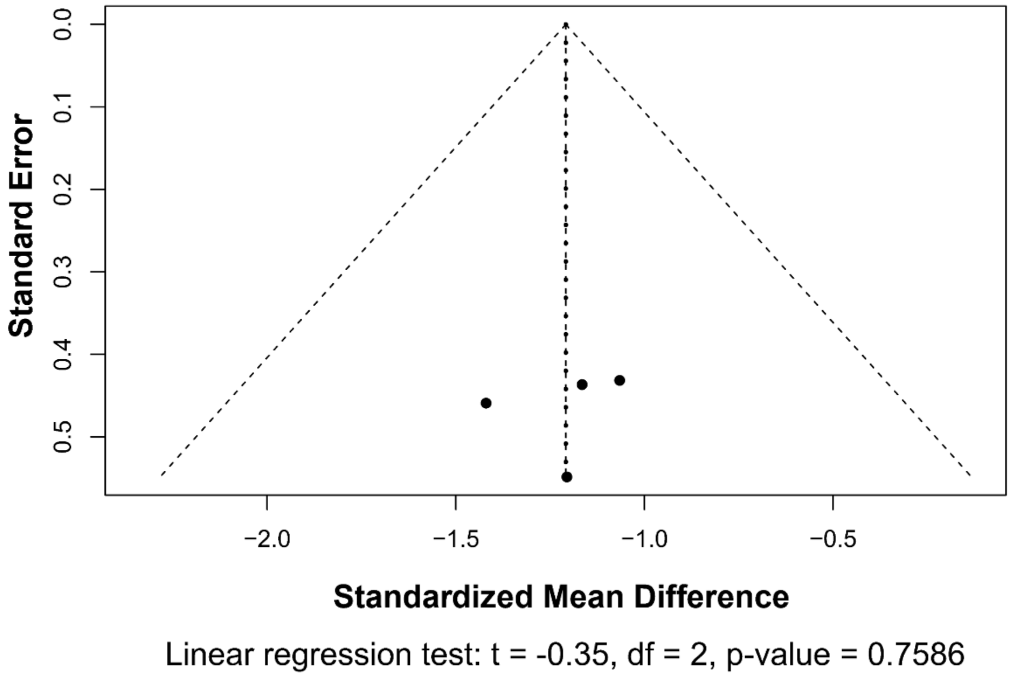

Supplementary Fig. 15

Ulcer index

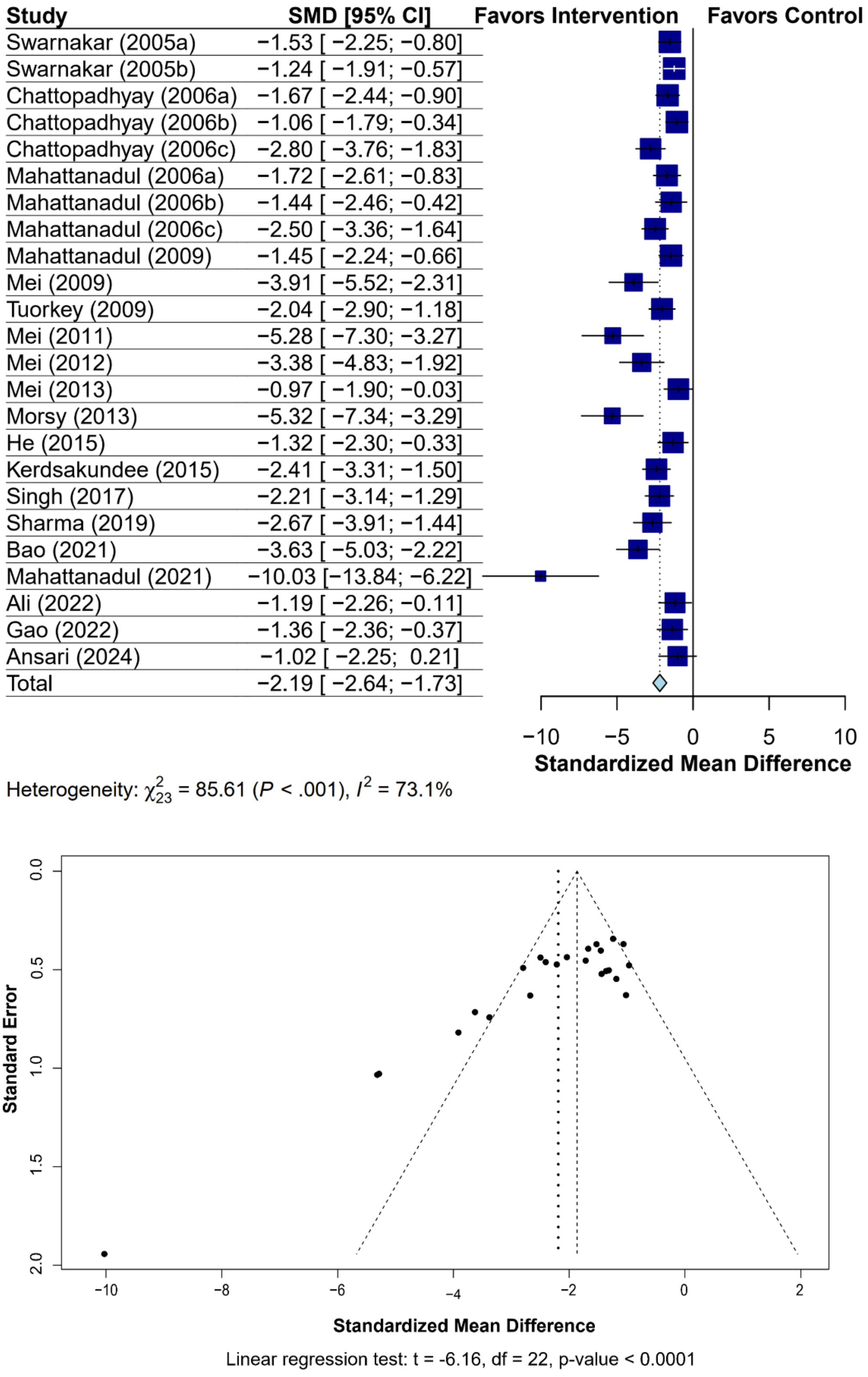

Supplementary Fig. 16

Mucosa healing index

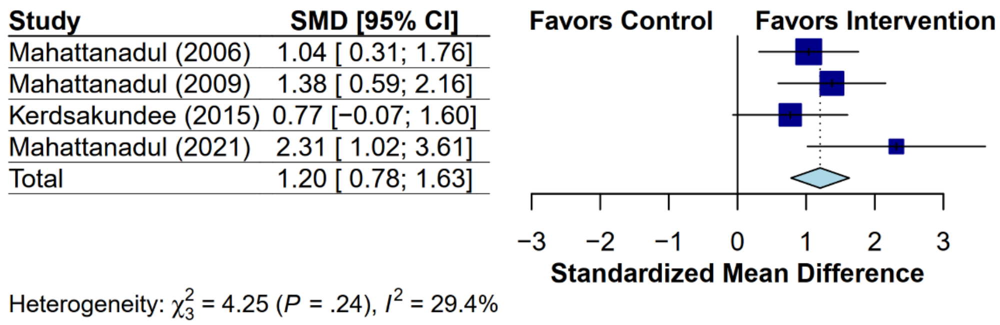

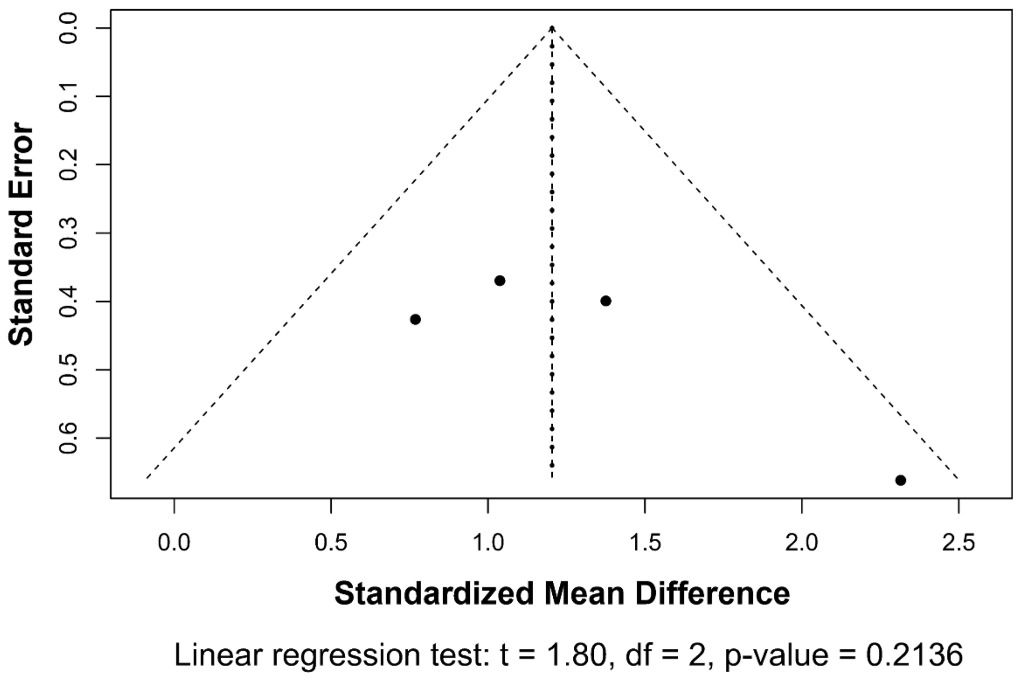

Supplementary Fig. 17

Ulcer healing index

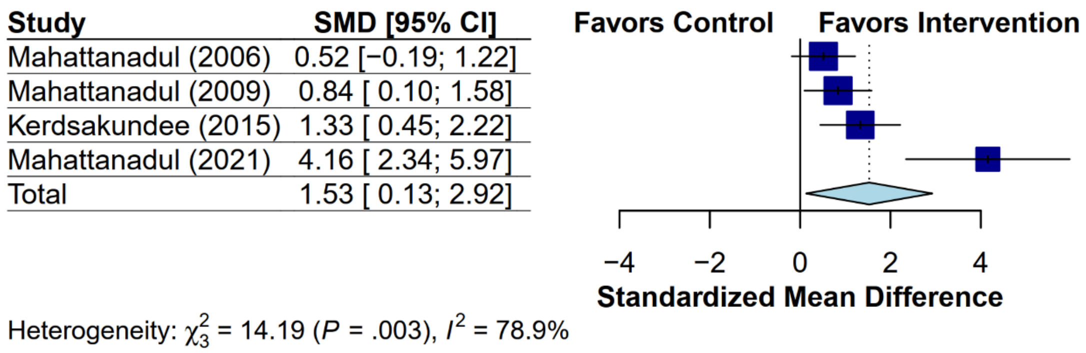

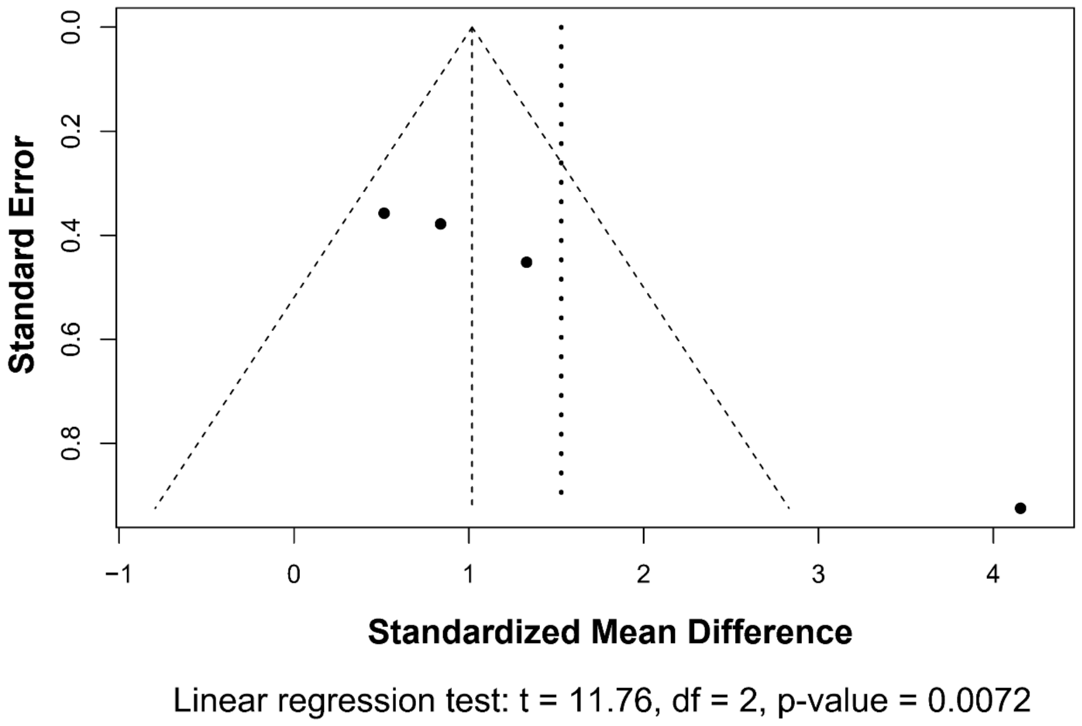

Supplementary Fig. 18

Blood glucose level

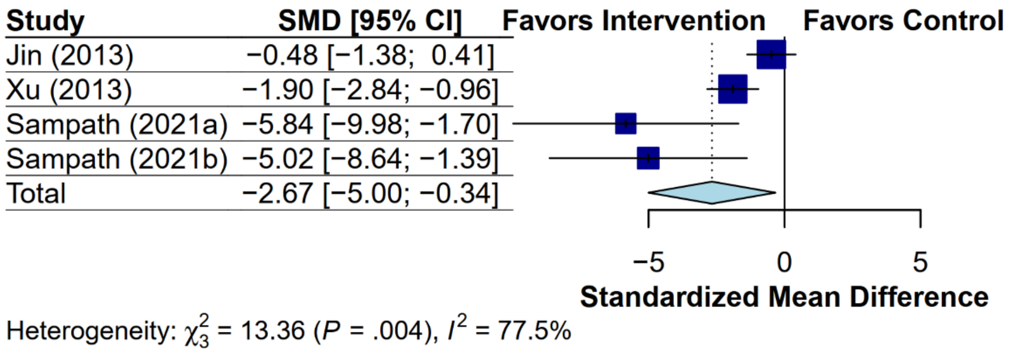

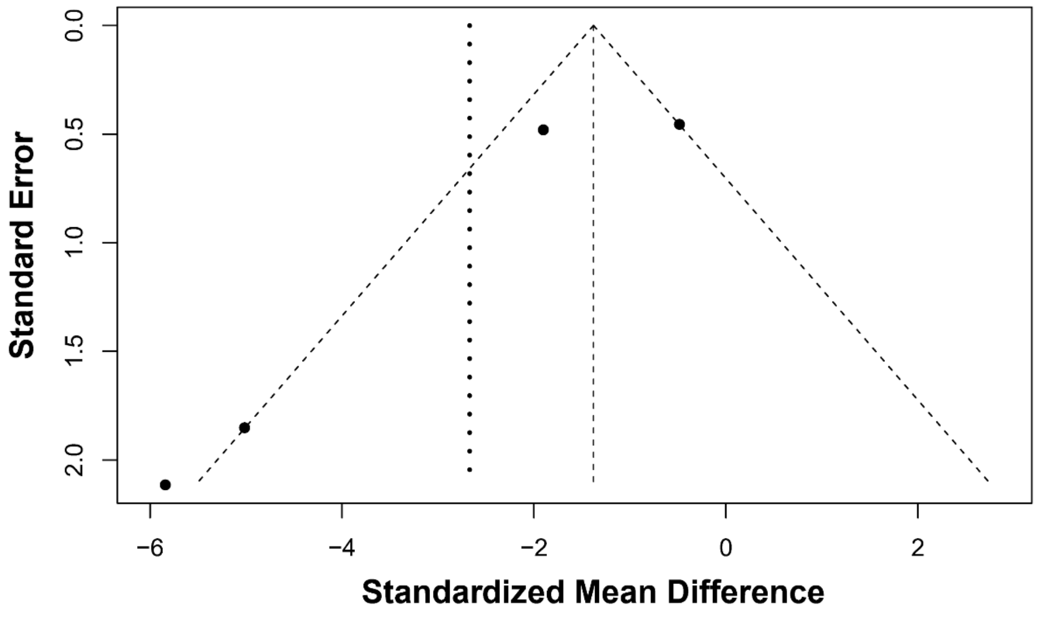

Linear regression test: t = -2.11, df = 2, p-value = 0.1693

Supplementary Fig. 19

Gastric emptying rate

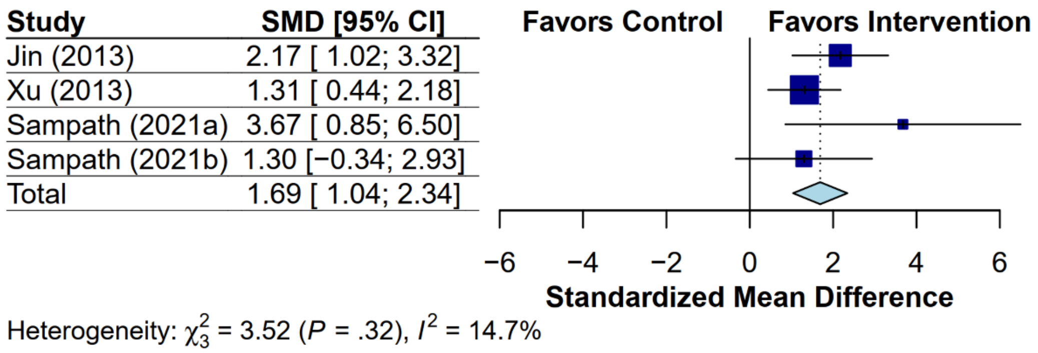

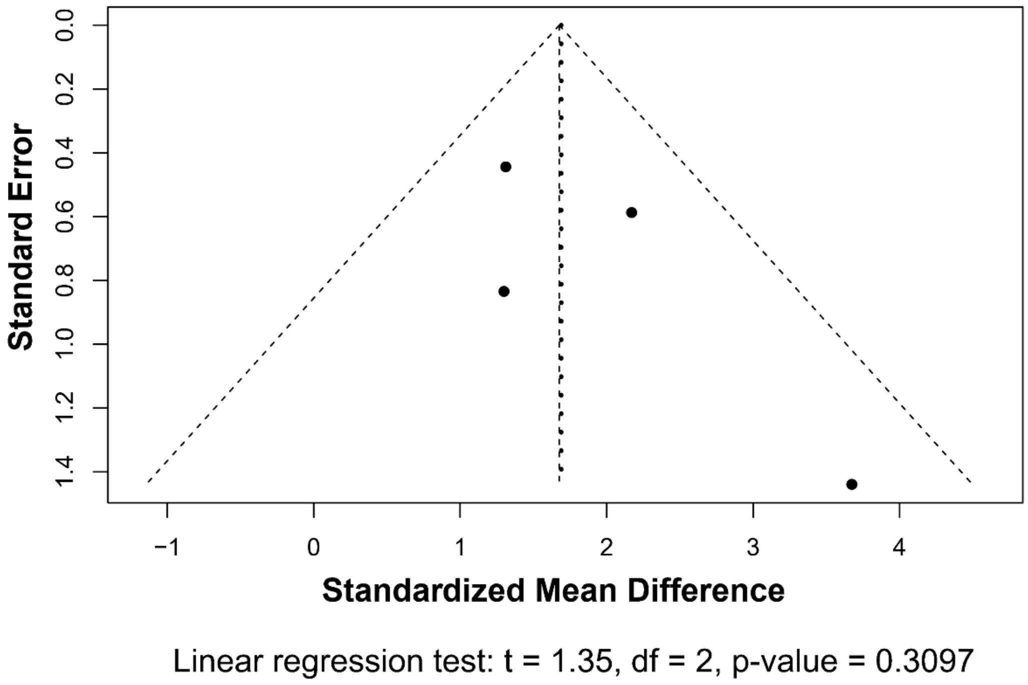

Supplementary Fig. 20

First author (year)	Dose of curcumin (mg/kg)	Curcumin formulation	Parameters related to upper gastrointestinal disorder																																												
			Esophageal tissue							Gastric juice		Gastric tissue																								Serum											
			Esophageal coefficient	Esophageal perforation	Histopathological analysis	Histopathological scoring	Interruption of lamina propria	Neutrophil infiltration	Ulcer index	Pepsin activity	Total acidity	Activities of MMP-3 and gelatinases	AP-1 protein expression	Catalase level	COX-2 protein and gene expression	DNA-fragmentation	GSH level	*H. pylori* colonization	*H. pyroli* specific genes *UreB* and *NapA*	Histopathological analysis	Histopathological scoring	Inflammatory response and immunity encoding genes	Iκ-B protein expression	Load of *H. pylori*	Macromolecular leakage	MDA levels	MPO level	Number of capillary sprout	Number of fibroblast	Neutrophils infiltration	OLGA scoring	Percentage of NF-κB p65 immunoreactive cells	PPAR-γ protein expression	Pro-inflammatory cyokines protein expression	SOD level	ALT level	AST level	IFN-γ level	Gastrin level	IgG level against *H. pyroli*	IgM level against *H. pyroli*	IL-4 level	LDH level	NO level	Sialic acid level	Somatostatin level	TAC level
**Esophageal disorder (n =4)**																																															
Mahattanadul (2006)	10	Suspension		↔					↔																																						
	20	Suspension		↓			↓	↓	↓	↓	↓																																				
	40	Suspension		↔					↔																																						
Sun (2019)	4.6	Suspension	↓		↔																																										
	eqv 3.8	Binary mixed micelle system	↓		R																																										
	eqv 4.0		↓		R																																										
Khwaldeh (2023)	40	Suspension			R																																										
Gurlek (2024)	100	Suspension				↔																																									
	200	Suspension			R	↓																														↓	↓						↓				
***H. pylori* infection (n =7)**																																															
De (2009)	25	Suspension																↓		R																											
Sintara (2010)	200	Suspension																↔			↓				↓							↓															
	600	Suspension																↔/ ↑*			↓				↓							↓															
Kundu (2011)	25	Suspension										↓	↓					↓	↓	R			↑										↑	↓													
	50	Suspension										↓						↓	↓	R																											
Ranjbar (2018)	200	Suspension																								↓	↓											↓	↓	↓	↔	↑				↔	
Santos (2018)	500	Lipidic solution													↓																																
Bendary (2023)	200	Clove oil Nanoemulsion																		R	↓																										
Mosallam (2023)	20 µg	Suspension																		R										↓																	
**Gastritis (n = 4)**																																															
Santos (2015)	500	Lipidic solution																		R		↓		↔																							
Hussein (2016)	100	Suspension												↑	↓	↓	↑			R							↓							↓	↑									↓	↓		
Gorzi (2021)	30	Suspension																		R						↓		↑	↓	↓																	↑
Halima (2023)	100	Suspension																		R										↓	↓																
	200	Suspension																		R										↓	↓																

Note: AP-1 = Activator protein-1, AST = Aspartate aminotransferase, COX-2 = Cyclooxygenase-2, CUR = curcumin, GSH = Glutathione, *H. pylori* = *Helicobacter pylori*, IgG = Immunoglobulin G, IgM = Immunoglobulin M, IFN-γ = Interferon-gamma, IL-4 = Interleukin-4, IκB = Inhibitor of kappa B, LDH = Lactate dehydrogenase, MDA = Malondialdehyde, MMP = Matrix metalloproteinase, MPO = Myeloperoxidase, n = number, NF-κB p65 = Nuclear factor kappa B p65, NO = nitric oxide, OLGA = Operative link for gastritis assessment, PPAR-γ = Peroxisome proliferator-activated receptor gamma, R = Restored, TAC = Total antioxidant capacity. * is present in mild colonization

in some areas and most gastric crypts. ↑ indicates an increase, ↓ indicates a decrease, and ↔ indicates no significant change in the parameter compared with the control group following curcumin intervention.

References

Al-Kawaz JM, Al-Harbi HJ, Al-Zubaidy FM. Therapeutic effects of esomeprazole, curcumin, chitosan, and Curcumin-Chitosan mixture on Ethanol-Induced gastric ulcer in female rats. J Angiother. 2024;8. https://doi.org/10.25163/angiotherapy.819439

Ali KA, El-Naa MM, Bakr AF, Mahmoud MY, Abdelgawad EM, Matoock MY. The dual gastro- and neuroprotective effects of curcumin loaded chitosan nanoparticles against cold restraint stress in rats. Biomed Pharmacother. 2022;148:112778. https://doi.org/10.1016/j.biopha.2022.112778

Ansari AZ, Doshi GM. Evaluation of the anti-ulcer activity of curcumin and linseed oil in an NSAID-induced gastric ulcer model in rats. Indian J Pharm Educ Res. 2024;58(Suppl 1):s252–s261. https://doi.org/10.5530/ijper.58.1s.27

Bao S, Zhang Y, Ye J, Zhu Y, Li R, Xu X, et al. Self-assembled micelles enhance the oral delivery of curcumin for the management of alcohol-induced tissue injury. Pharm Dev Technol. 2021;26(8):880–889. https://doi.org/10.1080/10837450.2021.1950185

Bendary MM, Elmanakhly AR, Mosallam FM, Alblwi NAN, Mosbah RA, Alshareef WA, et al. Boosting the anti-Helicobacter efficacy of azithromycin through natural compounds: Insights from in vitro, in vivo, histopathological, and molecular docking investigations. Helicobacter. 2024;29:e13110. https://doi.org/10.1111/hel.13110

Chattopadhyay I, Bandyopadhyay U, Biswas K, Maity P, Banerjee RK. Indomethacin inactivates gastric peroxidase to induce reactive-oxygen-mediated gastric mucosal injury and curcumin protects it by preventing peroxidase inactivation and scavenging reactive oxygen. Free Radic Biol Med. 2006;40(8):1397–1408. https://doi.org/10.1016/j.freeradbiomed.2005.12.016

Czekaj R, Majka J, Magierowska K, Sliwowski Z, Magierowski M, Pajdo R, et al. Mechanisms of curcumin-induced gastroprotection against ethanol-induced gastric mucosal lesions. J Gastroenterol. 2017;53:618–630. https://doi.org/10.1007/s00535-017-1385-3

Czekaj R, Majka J, Ptak-Belowska A, Szlachcic A, Targosz A, Magierowska K, et al. Role of curcumin in protection of gastric mucosa against stress-induced gastric mucosal damage. Involvement of hypoacidity, vasoactive mediators and sensory neuropeptides. J Physiol Pharmacol. 2016;67:261–275.

De R, Kundu P, Swarnakar S, Ramamurthy T, Chowdhury A, Nair GB, et al. Antimicrobial activity of curcumin against Helicobacter pylori isolates from India and during infections in mice. Antimicrob Agents Chemother. 2009;53:1592–1597. https://doi.org/10.1128/aac.01242-08

Díaz-Triste NE, González-García MP, Jiménez-Andrade JM, Castañeda-Hernández G, Chávez-Piña AE. Pharmacological evidence for the participation of NO–cGMP–KATP pathway in the gastric protective effect of curcumin against indomethacin-induced gastric injury in the rat. Eur J Pharmacol. 2014;731:102–106. https://doi.org/10.1016/j.ejphar.2014.02.030

Gandhi MN, Challa SR, Prasanth P, Gandhi TR. Role of leukotrienes in NSAID induced gastric ulceration and inflammation in Wistar rats. Asian Pac J Trop Dis. 2012;2:215–219. https://doi.org/10.1016/S2222-1808(12)60049-8

Gao J, Liu Y, Chen J, Tong C, Wang Q, Piao Y. Curcumin treatment attenuates cisplatin-induced gastric mucosal inflammation and apoptosis through the NF-κB and MAPKs signaling pathway. Hum Exp Toxicol. 2022;41. https://doi.org/10.1177/09603271221128738

Gorzi A, Asadi M, Voltarelli FA, Shamsi MM. Effects of curcumin on antioxidant capacity and gastric mucosal injury following strenuous endurance training in rats. Comp Exerc Physiol. 2020;17:17–24. https://doi.org/10.3920/cep200019

Gurlek IK, Muderrisoglu A, Er ZC, Arici A, Kupeli M. Evaluation of effects of curcumin on acute esophagitis in the corrosive esophagitis model in rats. Naunyn Schmiedebergs Arch Pharmacol. 2024;397:6677–6683. https://doi.org/10.1007/s00210-024-03038-2

Halima A, Akbar W, Ali H, Imtiaz H, Javed S. Residual level of deltamethrin induced gastritis and preventive role of curcumin on stomach mucosa. J Ayub Med Coll Abbottabad. 2023;35. https://doi.org/10.55519/jamc-04-11631

He P, Zhou R, Hu G, Liu Z, Jin Y, Yang G, et al. Curcumin-induced histone acetylation inhibition improves stress-induced gastric ulcer disease in rats. Mol Med Rep. 2014;11:1911–1916. https://doi.org/10.3892/mmr.2014.2958

Hussein SA, Karousa MM, Amin A, Awadalla MA. Curcumin ameliorates ethanol-induced gastric mucosal erosion in rats via alleviation of oxidative stress and regulation of pro-inflammatory cytokines and NF-κB activation. Nat Sci. 2016;4(4):466-76.

Ibrahim H, Metwaly E, Galal A, Sherif S. Potential curative effect of curcumin on gastric ulcer induced by piroxicam in male albino rats. Zagazig Vet J. 2019;47:378–387. https://doi.org/10.21608/zvjz.2019.14389.1056

Jin QH, Shen HX, Wang H, Shou QY, Liu Q. Curcumin improves expression of SCF/c-kit through attenuating oxidative stress and NF-κB activation in gastric tissues of diabetic gastroparesis rats. Diabetol Metab Syndr. 2013;5:12. https://doi.org/10.1186/1758-5996-5-12

Joshi A, Lehene S, Lasnapure B, Pawar S, Kandipati D, Panchal P. Investigation of antioxidant, anti-ulcer, and analgesic potential of a metal-curcumin complex. Naunyn Schmiedebergs Arch Pharmacol. 2023;396(5):1043-52. doi:10.1007/s00210-023-02407-5.

Kerdsakundee N, Mahattanadul S, Wiwattanapatapee R. Development and evaluation of gastroretentive raft forming systems incorporating curcumin-Eudragit® EPO solid dispersions for gastric ulcer treatment. Eur J Pharm Biopharm. 2015;94:513–20. https://doi.org/10.1016/j.ejpb.2015.06.024

Keçeci M, Ferah MA, Khoshvaghti H, Karaçetin S. Curcumin regulates inflammation and apoptosis through PARP-1 and NF-κB in ethanol-induced gastric ulcer model. Indian J Exp Biol. 2024;62(2):83-92.

Khwaldeh A. Protective effects of curcumin against nicotine-induced damage on esophagus tissue in the Wister rat. Trop J Nat Prod Res. 2023;7. https://doi.org/10.26538/tjnpr/v7i6.6

Kim JH, Jin S, Kwon HJ, Kim BW. Curcumin blocks naproxen-induced gastric antral ulcerations through inhibition of lipid peroxidation and activation of enzymatic scavengers in rats. J Microbiol Biotechnol. 2016;26:1392–7. https://doi.org/10.4014/jmb.1602.02028

Kuadkaew S, Ungphaiboon S, Phdoongsombut N, Kaewsuwan S, Mahattanadul S. Efficacy of a chitosan-curcumin mixture in treating indomethacin-induced acute gastric ulcer in rats. Curr Pharm Biotechnol. 2021;22:1919–31. https://doi.org/10.2174/1389201022666210127115427

Kundu P, De R, Pal I, Mukhopadhyay AK, Saha DR, Swarnakar S. Curcumin alleviates matrix metalloproteinase-3 and -9 activities during eradication of Helicobacter pylori infection in cultured cells and mice. PLoS One. 2011;6:e16306. https://doi.org/10.1371/journal.pone.0016306

Long L, Wang J, Chen N, Zheng S, Shi L, Xu Y, et al. Curcumin ameliorates reserpine-induced gastrointestinal mucosal lesions through inhibiting IκB-α/NF-κB pathway and regulating expression of vasoactive intestinal peptide and gastrin in rats. J Med Food. 2016;19:528–34. https://doi.org/10.1089/jmf.2015.3570

Mahattanadul S, Kongpuckdee S, Wiwattanapatapee R, Tansakul P, Nitiruangjaras A, Hansakul P. Comparative inhibitory efficacy on the iNOS/NO system of curcumin and tetrahydrocurcumin-self-microemulsifying liquid formulation in chronic gastric ulcer model. Curr Pharm Biotechnol. 2020;22:1005–12. https://doi.org/10.2174/1389201021666200807105849

Mahattanadul S, Nakamura T, Panichayupakaranant P, Phdoongsombut N, Tungsinmunkong K, Bouking P. Comparative antiulcer effect of bisdemethoxycurcumin and curcumin in a gastric ulcer model system. Phytomedicine. 2009;16:342–51. https://doi.org/10.1016/j.phymed.2008.12.005

Mahattanadul S, Reanmongkol W, Yano S, Panichayupakaranant P, Phdoongsombut N, Tungsinmunkong K. Preventive and curative effects of curcumin on the development of gastric inflammatory diseases in rats. J Nat Med. 2006;60:191–7. https://doi.org/10.1007/s11418-006-0035-5

Mahattanadul S, Radenahmad N, Phadoongsombut N, Chuchom T, Panichayupakaranant P, Yano S, Reanmongkol W. Effects of curcumin on reflux esophagitis in rats. J Nat Med. 2006;60(3):198-205. doi:10.1007/s11418-006-0036-4.

Mei X, Xu D, Wang S, Xu S. Pharmacological researches of curcumin solid dispersions on experimental gastric ulcer. Chin J Chin Mater Med. 2009;34:2920–3.

Mei X, Xu D, Xu S, Zheng Y, Xu S. Gastroprotective and antidepressant effects of a new zinc(II)–curcumin complex in rodent models of gastric ulcer and depression induced by

stresses. Pharmacol Biochem Behav. 2011;99:66–74. https://doi.org/10.1016/j.pbb.2011.04.002

Mei X, Xu D, Xu S, Zheng Y, Xu S. Novel role of Zn(II)–curcumin in enhancing cell proliferation and adjusting proinflammatory cytokine-mediated oxidative damage of ethanol-induced acute gastric ulcers. Chem Biol Interact. 2012;197:31–9. https://doi.org/10.1016/j.cbi.2012.03.006

Mei XT, Xu DH, Xu SK, Zheng YP, Xu SB. Zinc(II)–curcumin accelerates the healing of acetic acid-induced chronic gastric ulcers in rats by decreasing oxidative stress and downregulation of matrix metalloproteinase-9. Food Chem Toxicol. 2013;60:448–54. https://doi.org/10.1016/j.fct.2013.07.075

Morsy MA, El-Moselhy MA. Mechanisms of the protective effects of curcumin against indomethacin-induced gastric ulcer in rats. Pharmacology. 2013;91:267–74. https://doi.org/10.1159/000350190

Mosallam FM, Bendary MM, Elshimy R, El-Batal AI. Curcumin clarithromycin nano-form: a promising agent to fight Helicobacter pylori infections. World J Microbiol Biotechnol. 2023;39(12):324. doi:10.1007/s11274-023-03776-4.

Ranjbar R, Mohammadi A. Synergistic effects of combined curcumin and antibiotic in ameliorating an animal model of Helicobacter pylori infection. Biomed Res (India). 2018;29. https://doi.org/10.4066/biomedicalresearch.29-18-277

Sampath C, Wilus D, Tabatabai M, Freeman ML, Gangula PR. Mechanistic role of antioxidants in rescuing delayed gastric emptying in high fat diet induced diabetic female mice. Biomed Pharmacother. 2021;137:111370. https://doi.org/10.1016/j.biopha.2021.111370

Santos A, Lopes T, Oleastro M, Gato I, Floch P, Benejat L, et al. Curcumin inhibits gastric inflammation induced by Helicobacter pylori infection in a mouse model. Nutrients. 2015;7:306–20. https://doi.org/10.3390/nu7010306

Santos AM, Lopes T, Oleastro M, Pereira T, Alves CC, Seixas E, et al. Cyclooxygenase inhibition with curcumin in Helicobacter pylori infection. Nutrire. 2018;43. https://doi.org/10.1186/s41110-018-0070-5

Sharma V, Pathak K. Investigating gastroprotective potential of liquisolid curcumin against the role of endogenous aggressive factors and oxidative stress markers. Indian J Pharm Educ Res. 2019;53:527–36. https://doi.org/10.5530/ijper.53.3.85

Singh DP, Borse SP, Rana R, Nivsarkar M. Curcumin, a component of turmeric, efficiently prevents diclofenac sodium-induced gastroenteropathic damage in rats: a step towards translational medicine. Food Chem Toxicol. 2017;108:43–52. https://doi.org/10.1016/j.fct.2017.07.034

Sintara K. Curcumin suppresses gastric NF-κB activation and macromolecular leakage in Helicobacter pylori-infected rats. World J Gastroenterol. 2010;16:4039. https://doi.org/10.3748/wjg.v16.i32.4039

Sun Y, Li Y, Shen Y, Wang J, Tang J, Zhao Z. Enhanced oral delivery and anti-gastroesophageal reflux activity of curcumin by binary mixed micelles. Drug Dev Ind Pharm. 2019;45:1444–50. https://doi.org/10.1080/03639045.2019.1628041

Swarnakar S, Ganguly K, Kundu P, Banerjee A, Maity P, Sharma AV. Curcumin regulates expression and activity of matrix metalloproteinases 9 and 2 during prevention and healing of indomethacin-induced gastric ulcer. J Biol Chem. 2004;280:9409–15. https://doi.org/10.1074/jbc.m413398200

Thong-Ngam D. Curcumin prevents indomethacin-induced gastropathy in rats. World J Gastroenterol. 2012;18:1479. https://doi.org/10.3748/wjg.v18.i13.1479

Tuorkey M, Karolin K. Anti-ulcer activity of curcumin on experimental gastric ulcer in rats and its effect on oxidative stress/antioxidant, IL-6 and enzyme activities. Biomed Environ Sci. 2009;22:488–95. https://doi.org/10.1016/s0895-3988(10)60006-2

Xu L, Li Z, Guo F. Curcumin improves expression of ghrelin through attenuating oxidative stress in gastric tissues of streptozotocin-induced diabetic gastroparesis rats. Eur J Pharmacol. 2013;718:219–25. https://doi.org/10.1016/j.ejphar.2013.09.026
